# Systems Biology in Chronic Heart Failure—Identification of Potential miRNA Regulators

**DOI:** 10.3390/ijms232315226

**Published:** 2022-12-03

**Authors:** Alba Vilella-Figuerola, Alex Gallinat, Rafael Escate, Sònia Mirabet, Teresa Padró, Lina Badimon

**Affiliations:** 1 Cardiovascular-Program-ICCC, IR-Hospital Santa Creu i Sant Pau, IIB-Sant Pau, 08025 Barcelona, Spain; 2 Biochemistry and Molecular Biology Department, Universitat Autònoma de Barcelona (UAB), 08193 Cerdanyola del Vallès, Spain; 3 Centro de Investigación Biomédica en Red Cardiovascular (CIBER-CV), Instituto de Salud Carlos III, 28029 Madrid, Spain; 4 Heart Failure Group, Cardiology Department, Hospital Santa Creu i Sant Pau, 08025 Barcelona, Spain; 5 Chair Cardiovascular Research, Universitat Autònoma de Barcelona (UAB), 08025 Barcelona, Spain

**Keywords:** miRNA, chronic heart failure, network, enrichment pathways, pathophysiology

## Abstract

Heart failure (HF) is a complex disease entity with high clinical impact, poorly understood pathophysiology and scantly known miRNA-mediated epigenetic regulation. We have analysed miRNA patterns in patients with chronic HF (cHF) and a sex- and age-matched reference group and pursued an in silico system biology analysis to discern pathways involved in cHF pathophysiology. Twenty-eight miRNAs were identified in cHF that were up-regulated in the reference group, and eight of them were validated by RT-qPCR. In silico analysis of predicted targets by STRING protein-protein interaction networks revealed eight cluster networks (involving seven of the identified miRNAs) enriched in pathways related to cell cycle, Ras, chemokine, PI3K-AKT and TGF-β signaling. By ROC curve analysis, combined probabilities of these seven miRNAs (*let-7a-5p*, *miR-107*, *miR-125a-5p*, *miR-139-5p*, *miR-150-5p*, *miR-30b-5p* and *miR-342-3p*; clusters 1–4 [C:1–4]), discriminated between HF with preserved ejection fraction (HFpEF) and HF with reduced ejection fraction (HFrEF), and ischaemic and non-ischaemic aetiology. A combination of *miR-107*, *miR-139-5p* and *miR-150-5p*, involved in clusters 5 and 7 (C:5+7), discriminated HFpEF from HFrEF. Pathway enrichment analysis of miRNAs present in C:1–4 (*let-7a-5p*, *miR-125a-5p*, *miR-30b-5p* and *miR-342-3p*) revealed pathways related to HF pathogenesis. In conclusion, we have identified a differential signature of down-regulated miRNAs in the plasma of HF patients and propose novel cellular mechanisms involved in cHF pathogenesis.

## 1. Introduction

Heart failure (HF), affecting 1–2% of the world’s population over the age of 65, is one of the leading causes of mortality and morbidity [1,2]. It is a complex disease where the heart is unable to meet the metabolic demands of the body [3,4]. Chronic HF (cHF) has several underlying aetiologies (ischaemic disease, dilated cardiomyopathy, hypertrophy cardiomyopathy, hypertension, heart valve disease, etc.) that produce alterations in heart structure (left ventricle hypertrophy or dilation, cardiac fibrosis, heart remodelling) and function (abnormal cardiac output, diastolic dysfunction), leading to common symptomatology [3]. The underlying pathological mechanisms that conduce to cHF are not completely understood, but several biological pathways are involved in the progression of the disease [5].

Plasma sample analysis allows the easy acquisition of biologic material, such as microRNAs (miRNAs) that may assist in disease characterisation and patient risk stratification. Further, some plasma miRNAs provide prognosis information [6,7,8] (such as HF development after AMI [6]), or help in disease diagnosis [9,10], (as biomarkers of childhood dilated cardiomyopathy [9]). miRNAs are short RNA sequences (18–22 nucleotides long) that are involved in the regulation and control of RNA expression [11]. Given their role as transcriptional regulators, miRNAs are being thoroughly studied in several conditions [12]. Because of their stability and accessibility in blood, there is a great interest to determine the miRNA role in HF.

Some miRNAs have been related to cardiovascular (CV) disease pathogenesis [13,14] and the potential of others (e.g., *miR-423-5p*) as biomarkers for HF has been explored [15]. In that sense, we have previously determined that, in familial hypercholesterolaemia patients, up-regulated expression of miRNA *miR-133a* was associated with CV disease clinical event presentation. Further, we detected that this miRNA was mechanistically associated with atherosclerosis development [16]. Using a similar approach, we have investigated whether plasma miRNAs could show a differential pattern of expression in patients with cHF. Further, we performed an *in silico* systems biology analysis to predict protein targets of the identified differential miRNA signature and highlight those proteins involved in biological processes and molecular pathways with a higher probability to contribute to the pathophysiology of chronic heart failure.

## 2. Results

### 2.1. Differential miRNA Signature in cHF

**Figure 1:**
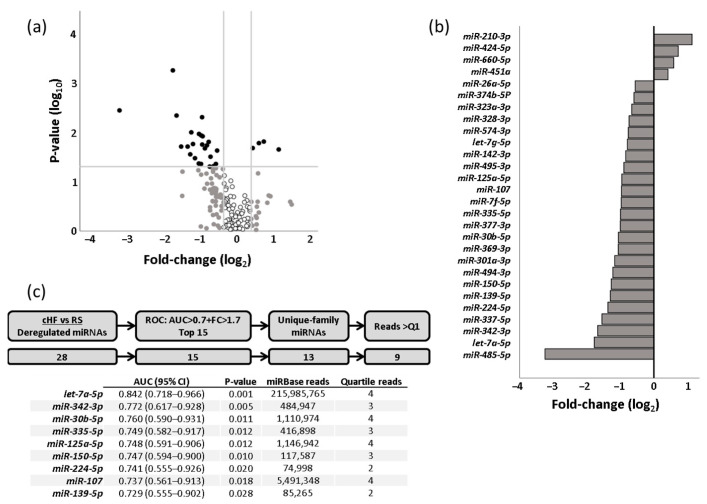
miRNA profiling in cHF patients and reference subjects. (**a**) miRNA profiling as a dot plot in a Cartesian graph. *x*-axis represents log_2_ (fold-change) and *y*-axis represents log_10_ (median concentration *p*-value). miRNAs with 1.3-fold changes in concentration level (greater than 0.38 [log_2_ (1.3)] and less than 0.38 [log_2_ (1/1.3)]) and significant *p*-value are shown in solid black dots. miRNAs with 1.3-fold changes in concentration level (greater than 0.38 [log_2_ (1.3)] and less than 0.38 [log_2_ (1/1.3)]) but non-significant *p*-value are shown in solid grey dots. miRNAs with less than 1.3-fold changes in concentration level (lower than 0.38 [log_2_ (1.3)] and higher than 0.38 [log_2_ (1/1.3)]) and non-significant *p*-value are shown in black circles. (**b**) Bar graph indicating changes in the expression of miRNAs between patients and reference subjects. (**c**) Schematic workflow for miRNA selection. AUC: area under the curve; cHF: chronic heart failure; FC: fold-change; Q1: first (lowest) quartile; RS: reference subjects; ROC: receiver operating characteristic curve.

We comparatively analysed the plasma levels of 377 miRNAs in cHF patients and reference subjects (RS). Of these 377 miRNAs, 167 were consistently detected in both groups. Differential expression analyses between cHF and RS showed that 28 of these miRNAs presented statistically significant differences and a fold-change (log_2_) higher than ±0.38 between groups. Of these, the top 15 miRNAs presenting an area under the curve (AUC) ≥0.7 after C-statistics analysis and a fold-change >1.7 (0.75 in log_2_) were selected for further analysis. Three of the top-fifteen miRNAs were of the same family (*let-7a-5p*, *let-7f-5p* and *let-7g-5p*) and have been reported to be involved in the regulation of common pathways [17]. Therefore, only miRNA *let-7a-5p*, with the best AUC among them, was further analysed. In addition, and considering the probability of a low detection level, we excluded those miRNAs that according to miRBase presented a number of reads in the lowest 25% range (Figure 1 and Supplemental Table S1).

Thus, nine miRNAs (*let-7a-5p*, *miR-107*, *miR-125a-5p*, *miR-139-5p*, *miR-150-5p*, *miR-224-5p*, *miR-30b-5p*, *miR-335-5p* and *miR-342-3p*) were further selected for validation. Additionally, we selected two miRNAs (*miR-16-5p* and *miR-223-3p*) that had similar expression values between groups as negative controls.

**Figure 2:**
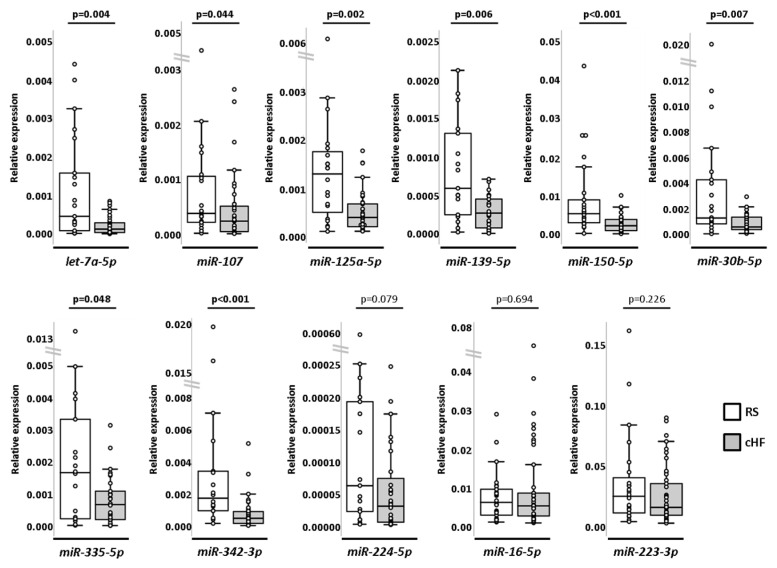
Box and whisker plots of selected miRNAs analysed in cHF (*n* = 46) and RS (*n* = 26). U Mann–Whitney test. A *p*-value < 0.05 was considered significant. cHF: chronic heart failure; RS: reference subjects.

These selected miRNAs were further validated by RT-qPCR using customised cards in larger cHF (*n* = 46) and RS (*n* = 26) groups. Differences between groups reached statistical power in eight miRNAs (*let-7a-5p*, *miR-107*, *miR-125a-5p*, *miR-139-5p*, *miR-150-5p*, *miR-30b-5p*, *miR-335-5p* and *miR-342-3p*), being all down-regulated in cHF compared to RS. Differential expression of *miR-224-5p*, although showing a similar trend, did not reach statistical significance. Negative controls (*miR-16-5p* and *miR-223-3p*) maintained similar expression between groups (Figure 2 and Supplemental Table S2).

### 2.2. In Silico System Biology Analysis and Target Prediction

Gene-targets of the differential miRNA signature (577 genes) were obtained with the combination of four different miRNA-target prediction algorithms (TargetScan, miRmap, miRWalk and miRDB [18,19,20,21,22]). All validated miRNAs were included in target data mining (*let-7a-5p*, *miR-107*, *miR-125a-5p*, *miR-139-5p*, *miR-150-5p*, *miR-30b-5p* and *miR-342-3p*), except *miR-335-5p*, given that, for this miRNA, only one target was obtained after combining predictions of all four algorithms used. The resulting targets were analysed using the ShinyGO web-based software [23] for pathway enrichment analysis (Table 1). Gene ontology (GO) annotations for biological processes of predicted targets, sorted by average and rank (FDR and fold), revealed significant enrichment in pathways related to coronary vasculature morphogenesis and development (GO:0060977 and GO:0060976, respectively; FDR < 0.001, both), TGF-β signalling regulation (GO:0030511 and GO:1903846; FDR < 0.001, both) and with ventricular and cardiac septum development (GO:0003281 and GO:0003279, respectively; FDR < 0.001, both).

**Figure 3:**
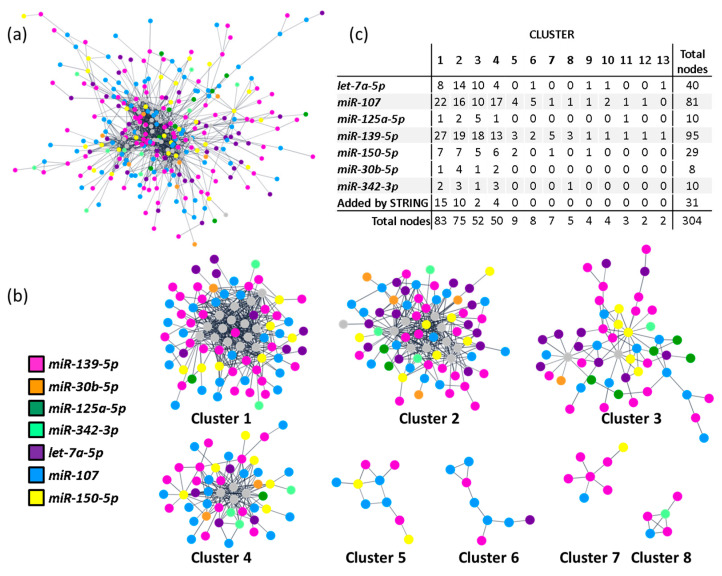
miRNA clustering and enrichment analysis. (**a**) PPI network of the circulating miRNA signature in cHF. Only interactions with a confidence score higher than 0.8 were considered. Node colours represent targeting miRNA. Grey nodes correspond to STRING-added nodes. (**b**) Community clusters identified within the main connected component of the PPI network. Clusters with less than 5 genes were excluded. (**c**) Table with number of target genes regulated by each miRNA, per cluster. cHF: chronic heart failure; PPI: protein-protein interaction.

Additionally, a protein-protein interaction (PPI) network, including all 577 predicted targets, was built according to data from the STRING database [24] using Cytoscape [25]. The resulting network revealed a highly connected nodule involving 273 targets from all included miRNAs (Figure 3a). We then applied a community cluster strategy to identify PPI-enriched nodules [26] and ran a functional overrepresentation analysis upon pathways from the KEGG database [27,28]. Clusters with fewer than five nodes were excluded from the analysis. As a result, eight clusters were identified from the main connected component of the network (Figure 3b). Noteworthy, four clusters included targets from all query miRNAs (clusters 1–4) and were the ones selected for more specific pathway analysis. As depicted in Supplemental Table S3, the identified clusters aggregate functionally related genes. Highly significant enrichment for focal adhesions (hsa04510; FDR < 0.001), chemokine, Ras and PI3K-AKT signalling pathways (hsa04062, hsa04014 and hsa04151, respectively; FDR < 0.001, all) was found for cluster 1. Also, strong enrichments in cell cycle (hsa04110; FDR < 0.001), cellular senescence (hsa04218; FDR < 0.001) and TGF-β signalling (hsa04350; FDR < 0.001) were detected for cluster 2. For clusters 3 and 4, only marginal enrichments were found in genes involved in RNA transport (hsa03013; FDR < 0.001), Wnt and Hippo signalling pathways (hsa04310 and hsa04390, respectively; FDR < 0.001).

### 2.3. Association of Clusters with cHF Subtypes

Considering that the networks obtained in the PPI analysis associated with specific pathways, we analysed if clustered miRNAs provided new information to differentiate between HF subtypes. As shown in Table 2, we observed that C-statistics analysis of the combined probabilities of those miRNAs with a role in clusters 1–4 (including miRNAs *let-7a-5p*, *miR-107*, *miR-125a-5p*, *miR-139-5p*, *miR-150-5p*, *miR-30b-5p* and *miR-342-3p*; from now on “C:1–4”) differentiated between patients with HF with preserved ejection fraction (HFpEF) and HF with reduced ejection fraction (HFrEF) (AUC: 0.725 ± 0.102 [95% Confidence Interval (CI): 0.526–0.925]; *p* = 0.047) and between cHF underlying aetiologies (ischaemic vs. non-ischaemic; AUC: 0.789 ± 0.088 [95% CI: 0.617–0.962]; *p* = 0.019), but not need for rehospitalisation (*p* = 0.880).

Similarly, the combined probabilities of miRNAs in clusters 5 and 7 (*miR-107*, *miR-139-5p* and *miR-150-5p*; from now on “C:5+7”) could differentiate between HFpEF and HFrEF (AUC: 0.782 ± 0.083 [95% CI: 0.618–0.945]; *p* = 0.007), but not between background aetiologies (*p* = 0.216). These miRNAs presented a tendency to discriminate the need for rehospitalisation, but it did not reach statistical significance (*p* = 0.080).

The combined probabilities of miRNAs involved in cluster 6 (*miR-107*, *miR-139-5p* and *let-7a-5p*) did not differentiate between ejection fractions (*p* = 0.467), underlying aetiology (*p* = 0.124) or need for rehospitalisation (*p* = 0.228). The combination of miRNAs included in cluster 8 (*miR-107*, *miR-139-5p* and *miR-342-3p*) presented a borderline tendency to differentiate between cHF ejection fraction groups (*p* = 0.068) and the need for rehospitalisation (*p* = 0.074). They could not, however, discriminate between background aetiologies (*p* = 0.193).

To study if the miRNAs implicated in the clusters with discriminatory potential associated with specific pathways, we performed two other PPI networks. Specifically, we compared if the miRNAs included in C:5+7 (*miR-107*, *miR-139-5p* and *miR-150-5p*) and the miRNAs not included in these clusters but present in C:1–4 (*let-7a-5p*, *miR-125a-5p*, *miR-30b-5p* and *miR-342-3p*; from now on “Differential miRNAs [DiffmiR]”) were enriched in different biological pathways. Further, we analysed if those divergent pathways could be associated with the discriminatory differences observed between C:1–4 and C:5+7.

**Figure 4:**
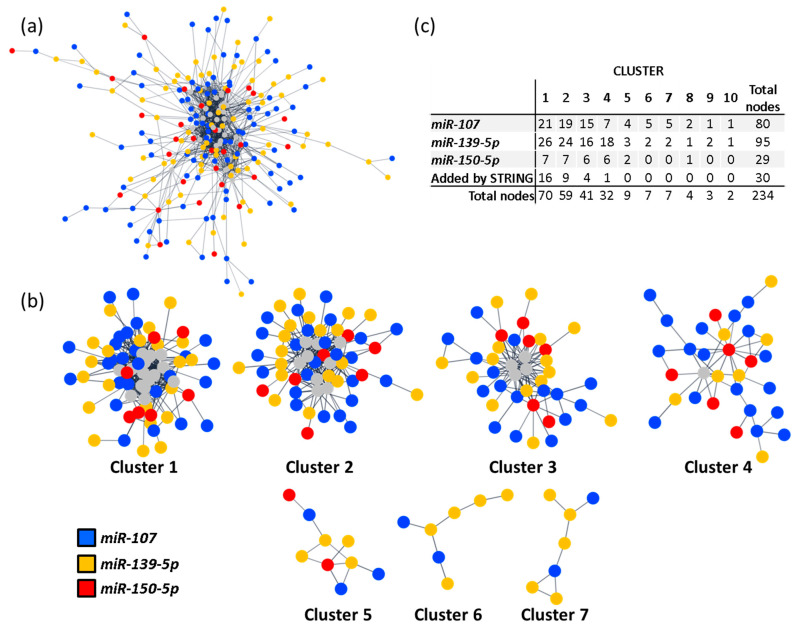
miRNA in C:5+7 clustering and enrichment analysis. (**a**) PPI network of the miRNAs included in clusters 5 and 7 (C:5+7) of the main PPI network. Only interactions with a confidence score higher than 0.8 were considered. Node colour represents targeting miRNA. Grey nodes correspond to STRING-added nodes. (**b**) Community clusters identified within the main connected component of the C:5+7 PPI network. Clusters with fewer than five genes were excluded. (**c**) Table with number of target genes regulated by each miRNA, per cluster. C:5+7: miRNAs included in clusters 5 and 7 of the main PPI network (includes *miR-107*, *miR-139-5p* and *miR-150-5p*); PPI: protein-protein interaction.

**Figure 5:**
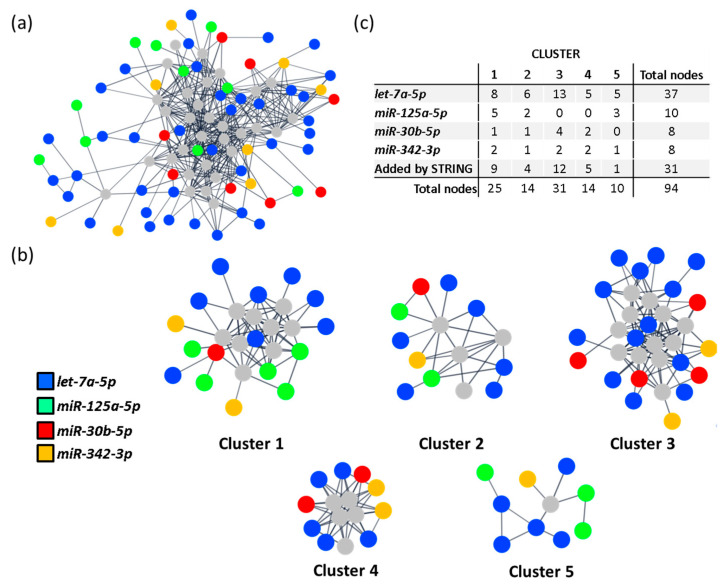
miRNA in DiffmiR clustering and enrichment analysis. (**a**) PPI network of the miRNAs included in C:1–4 but not in C:5+7 of the main PPI network (DiffmiR). Only interactions with a confidence score higher than 0.8 were considered. Node colour represents targeting miRNA. Grey nodes correspond to STRING-added nodes. (**b**) Community clusters identified within the main connected component of the DiffmiR network. Clusters with fewer than five genes were excluded. (**c**) Table with number of target genes regulated by each miRNA, per cluster. C:1–4: miRNAs included in clusters 1–4 of the main PPI network (includes *let-7a-5p*, *miR-107*, *miR-125a-5p*, *miR-139-5p*, *miR-150-5p*, *miR-30b-5p* and *miR-342-3p*); C:5+7: miRNAs included in clusters 5 and 7 of the main PPI network (includes *miR-107*, *miR-139-5p* and *miR-150-5p*); DiffmiR: miRNAs included in clusters 1–4 but not in clusters 5 and 7 of the main PPI network (includes *let-7a-5p*, *miR-125a-5p*, *miR-30b-5p* and *miR-342-3p*); PPI: protein-protein interaction.

Thus, two PPI networks were generated in Cytoscape with data from STRING [24]. Networks for C:5+7 and DiffmiR were created with 408 and 189 predicted target genes, respectively. The new resulting networks included 204 predicted targets for C:5+7 and 63 for DiffmiR (Figure 4a and Figure 5a). To identify PPI-enriched nodules, we applied a community cluster strategy [26], as described above, and pursued a functional overrepresentation analysis upon pathways from the KEGG database [27,28]. Only clusters with more than five nodes were considered for analysis.

As a result, seven clusters were generated from the main connected component of the C:5+7 network (Figure 4b). Of these, five clusters included targets from the three involved miRNAs and were selected for pathway enrichment analysis (Supplemental Table S4). Cluster 1 was significantly enriched in focal adhesions (hsa04510; FDR < 0.001), and Ras, chemokine and PI3K-AKT signalling pathways (hsa04014, hsa04062 and hsa04151, respectively; FDR < 0.001 all). Cluster 2 was enriched in Notch, TGF-β and thyroid hormone signalling pathways (hsa04330, hsa04350 and hsa04919, respectively; FDR < 0.001, all), as well as cell cycle (hsa04110; FDR < 0.001). Cluster 3 was enriched in pathways related to ubiquitin-mediated proteolysis (hsa04120; FDR < 0.001), and Wnt and Hippo signalling pathways (hsa04310 and hsa04390, respectively; FDR < 0.0002, both). Clusters 4 and 5 were marginally enriched in RNA transport (hsa03013; FDR < 0.001) and taste transduction (hsa04742; FDR < 0.001).

From the main connected component of the DiffmiR, five clusters were generated (Figure 5b). Only those clusters with target genes of the four miRNAs included in the analysis were selected for pathway enrichment analysis (clusters 1–2). Cluster 1 was enriched in prolactin, PI3K-AKT, ErbB and JAK-STAT signalling pathways (hsa04917, hsa04151, hsa04012 and hsa04630, respectively; FDR < 0.001, all), as well as in EGFR tyrosine kinase inhibitor resistance (hsa01521; FDR < 0.001) and endocrine resistance (hsa01522; FDR < 0.001). Cluster 2 was enriched in cellular senescence (hsa04218; FDR < 0.001) and TGF-β signalling pathway (hsa04350; FDR < 0.001) (Supplemental Table S5).

**Figure 6:**
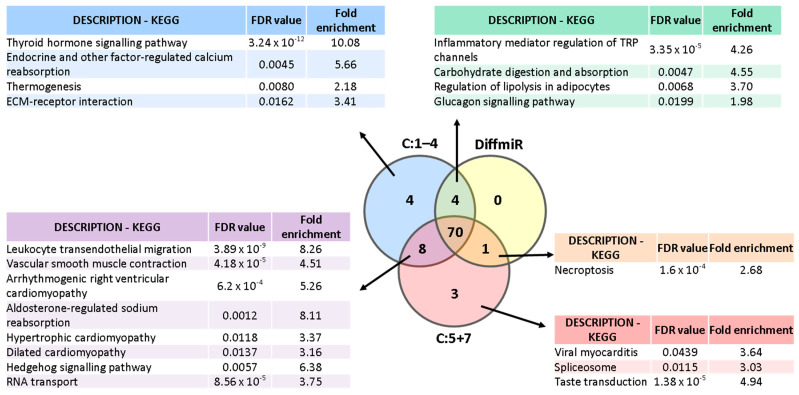
Venn diagram of enriched pathways identified for each group of miRNAs. C:1–4: miRNAs included in clusters 1–4 of the main PPI network (includes *let-7a-5p*, *miR-107*, *miR-125a-5p*, *miR-139-5p*, *miR-150-5p*, *miR-30b-5p* and *miR-342-3p*); C:5+7: miRNAs included in clusters 5 and 7 of the main PPI network (includes *miR-107*, *miR-139-5p* and *miR-150-5p*); DiffmiR: miRNAs included in clusters 1–4 but not in clusters 5 and 7 of the main PPI network (includes *let-7a-5p*, *miR-125a-5p*, *miR-30b-5p* and *miR-342-3p*).

These results were then compared with the pathways obtained in the pathway enrichment analysis of the PPI including all miRNAs (*let-7a-5p*, *miR-107*, *miR-125a-5p*, *miR-139-5p*, *miR-150-5p*, *miR-30b-5p* and *miR-342-3p*; Figure 3). Only those pathways unique to C:1–4, C:5+7 or DiffmiR, as well as common between C:1–4 and C:5+7/DiffmiR (Figure 6), were considered.

Thus, the C:5+7 network was enriched in leukocyte transendothelial migration (hsa04670; FDR < 0.001), vascular smooth muscle contraction (hsa04270; FDR < 0.001), arrhythmogenic right ventricular, hypertrophic and dilated cardiomyopathy (hsa05412, hsa05410, hsa05414, respectively; FDR < 0.02, all), aldosterone-regulated sodium reabsorption (hsa04960; FDR = 0.0012), viral myocarditis (hsa05416; FDR = 0.043), hedgehog signalling pathway (hsa04340; FDR = 0.0057), RNA transport (hsa03013; FDR < 0.001), spliceosome (hsa03040; FDR = 0.0115) and taste transduction (hsa04742; FDR < 0.001).

The DiffmiR network was enriched in glucagon signalling pathway (hsa04922; FDR = 0.019), inflammatory mediator regulation of TRP channels (hsa04750; FDR < 0.001), carbohydrate digestion and absorption (hsa04973; FDR = 0.0047) and regulation of lipolysis in adipocytes (hsa04923; FDR = 0.0068).

### 2.4. miR-107 and miR-139 Were Overrepresented in All Clusters

When the weight of each miRNA in each cluster of the main PPI network was considered (Figure 3a), we observed that *miR-107* and *miR-139-5p* were overrepresented, with 81 (26.4%) and 95 (31.25%) of all clustered protein-coding genes targeted by these miRNAs, respectively (Figure 3c). Therefore, *miR-107* and *miR-139-5p* were analysed individually to better understand their specific contribution to cHF pathophysiology.

In silico analysis querying “heart failure” to STRING Pubmed in Cytoscape [25] resulted in a repository-based list of 837 genes related to HF (repository-based gene database [RBGD]). The 81 and 95 targets of *miR-107* and *miR-139-5p*, respectively, were matched individually to the RBGD list (Supplemental Figure S1). Using this approach, we found three protein-coding genes regulated by *miR-107* and nine regulated by *miR-139-5p* that were also identified as genes with a role in HF, according to the repository-derived data included in the RBGD list (Table 3).

**Figure 7:**
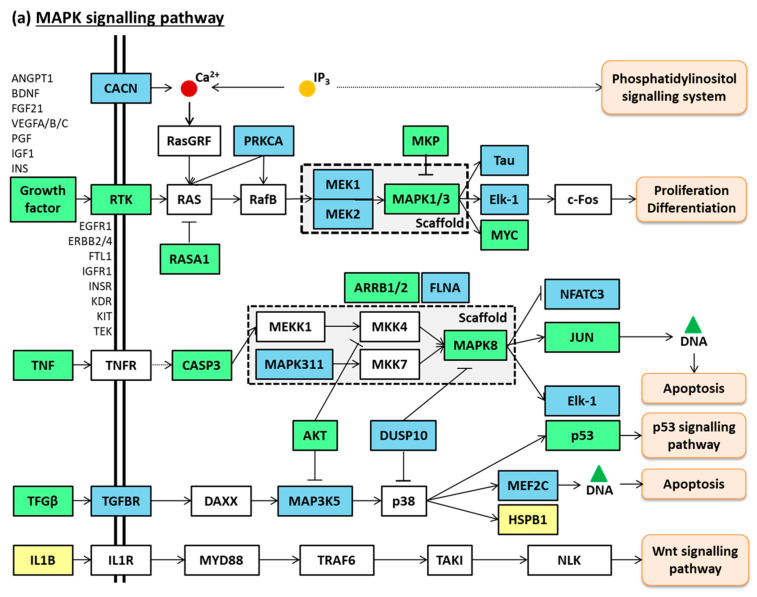

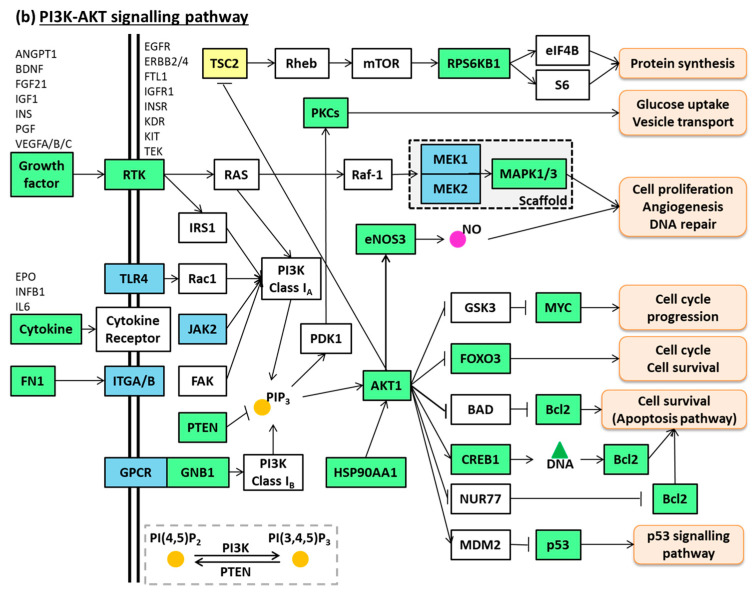
*miR-107* and *miR-139-5p* common pathways. (**a**) MAPK signalling pathway. (**b**) PI3K-AKT signalling pathway. Coloured nodes correspond to target genes and their neighbouring genes. In blue: *miR-139-5p* target genes and their neighbouring genes; in yellow: *miR-107* target genes and their neighbouring genes; in green: *miR-139-5p* and *miR-107* common target genes and their neighbouring genes; in white: additional nodes for pathway completion. Unframed gene names correspond to targeted genes included under a broader category such as “growth factor”, “RTK” or “cytokine”. Adapted from pathways “MAPK signalling pathway (hsa04010)” and “PI3K-AKT signalling pathway (hsa04151)” generated in the KEGG database.

These 12 common protein-coding genes and their RBGD-based neighbouring interacting genes (101 for *miR-107* and 176 for *miR-139-5p*) were selected and analysed with ShinyGO [23] to determine their putative pathways. Using the KEGG database [27,28], we observed that both miRNAs participated in the pathways PI3K-AKT signalling (hsa04151; FDR < 0.001; both miRNAs) and MAPK signalling (hsa04010; FDR < 0.001; both miRNAs) (Figure 7a,b).

### 2.5. Association of Individual Plasma miRNAs with Aetiology and with Left Ventricular Ejection Fraction in cHF Patients

**Figure 8:**
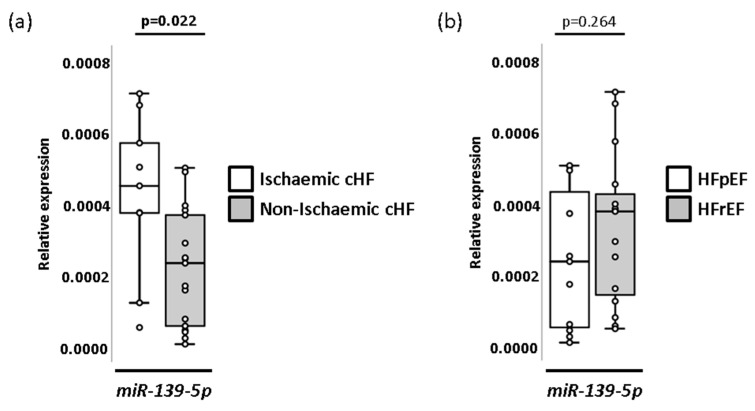
miRNAs patterns associations with cHF underlying aetiology and ejection fraction. (**a**) Box and whisker plots of miRNA *miR-139-5p* in cHF patients according to underlying aetiology (ischaemic and non-ischaemic). (**b**) Box and whisker plots of miRNA *miR-139-5p* in cHF patients according to ejection fraction (HFpEF and HFrEF). A *p <* 0.05 was considered significant. cHF: chronic heart failure; HFpEF: heart failure with preserved ejection fraction; HFrEF: heart failure with reduced ejection fraction.

A study of individual miRNAs showed that *miR-139-5p* presented differential expression patterns between cHF patients with and without underlying ischaemic aetiology (*p* = 0.022), but not for left ventricular ejection fraction (LVEF) levels (Figure 8 and Supplemental Tables S6 and S7). miRNA plasma levels did not show statistically significant changes between patients with different degrees of severity according to the New York Heart Association (NYHA) classification.

## 3. Discussion

Heart failure is a highly frequent disease with a complex pathophysiology [3]. Circulating miRNAs are emerging as tools to facilitate disease diagnosis, prognosis, risk stratification and management [6,7,8,9,10]. In addition, miRNAs have a role in gene expression regulation and are, therefore, involved in disease pathogenesis [12,13]. In this study, we have used a non-targeted approach to miRNA identification and investigated plasma samples of cHF patients. We have studied the putative role of miRNAs in cHF pathogenesis through gene regulation by pursuing an in silico enrichment pathway analysis. Additionally, we analysed if these miRNAs were individually related to pathways associated with cHF pathogenesis.

In this study, we have identified the presence of a down-regulated eight-miRNA pattern in the plasma of cHF patients in comparison to a group of reference subjects. Specifically, circulating levels of miRNAs *let-7a-5p*, *miR-107*, *miR-125a-5p*, *miR-139-5p*, *miR-150-5p*, *miR-30b-5p*, *miR-335-5p* and *miR-342-3p* were significantly lower in cHF than in the RS group. Predicted target genes of seven of these miRNAs (*let-7a-5p*, *miR-107*, *miR-125a-5p*, *miR-139-5p*, *miR-150-5p*, *miR-30b-5p* and *miR-342-3p*) clustered and were related to several pathways, including cell cycle, cellular senescence, or Ras, chemokine, PI3K-AKT and TGF-β signalling pathways. Interestingly, we found that, when miRNAs were considered according to their clusterisation, they had discriminative value. Specifically, we observed that the best discrimination for ejection fraction types (HFpEF and HFrEF) was obtained with those miRNAs regulating cluster 5 and 7 (*miR-107*, *miR-139-5p* and *miR-150-5p*; C:5+7), followed by those miRNAs involved in clusters 1–4 (*let-7a-5p*, *miR-107*, *miR-125a-5p*, *miR-139-5p*, *miR-150-5p*, *miR-30b-5p* and *miR-342-3p*; C:1–4). The latest miRNA combination also differentiated background aetiology, but the combination of *miR-107*, *miR-139-5p* and *miR-150-5p* (C:5+7) did not. For this reason, we explored if miRNAs in C:1–4 but not in C:5+7 were related to specific pathways that could explain this difference. Thus, the cluster involving *let-7a-5p*, *miR-125a-5p*, *miR-30b-5p* and *miR-342-3p* (DiffmiR), associated with metabolic (glucagon signalling pathway, lipolysis regulation and carbohydrate digestion and absorption) and inflammatory pathways (inflammatory mediator regulation of TRP channels). In contrast, miRNAs in the C:5+7 had gene targets involved in cardiomyopathy-related pathways (hypertrophic, dilated and arrhythmogenic cardiomyopathies), aldosterone-regulated sodium reabsorption, vascular smooth muscle cell contraction, or leukocyte trans-endothelial migration.

In contrast to our findings at the systemic level, *let-7a-5p* was found up-regulated in plasma from the coronary sinus of cHF patients when compared to controls [29]; although differences between both studies could relate to the plasmatic sample origin. Our study could reflect systemic changes in cHF, whereas those miRNA patterns in the coronary sinus could be more related to pathological alterations in the heart [29]. In agreement with our results, Ellis and colleagues [30] detected down-regulated levels of *miR-150-5p* in the plasma of cHF in comparison to controls [30]. Similarly, Scrutinio et al. [31] reported *miR-150-5p* to be down-regulated in cHF patients with severe symptomatology in comparison to cHF with mild symptomatology and to controls, being *miR-150-5p* associated with an increased risk of death, heart transplantation or left ventricular assist devices implantation [31]. Furthermore, the serum levels of *miR-150-5p* have been reported to be significantly decreased in acute myocardial infarction (AMI) patients compared to controls, with AMI patients who developed cHF presenting the lowest levels [32]. The same study suggested that *miR-150-5p* was able to predict post-AMI cHF when combined with brain natriuretic peptide (BNP)[32] and that *miR-150-5p* was independently associated with post-AMI cHF through multivariate analysis including age, BNP and LVEF [32]. Although we did find a negative correlation of this miRNA with LVEF, we did not observe an association of *miR-150-5p* with ischaemic disease. This could be due to the sample type (plasma instead of serum) [33], or to the fact that we compared those patients with cHF due to an AMI with those patients that developed cHF due to other reasons, such as heart valve disease or cardiac hypertrophy, which could also affect *miR-150-5p* levels.

In contrast, we observed that miRNA *miR-139-5p* was up-regulated in the plasma of those patients with underlying ischaemic disease in comparison with those with underlying non-ischaemic disease. Divergent results were found by Voellenkle et al., who observed similar levels of this miRNA (from peripheral blood mononuclear cells [PBMCs]) between cHF with ischaemic and non-ischaemic backgrounds [34]. Nevertheless, in agreement with our findings, the authors described down-regulated levels of *miR-139-5p* in cHF [34]. The difference in ischaemia-related *miR-139-5p* response between blood cells and plasma samples suggests that a tissue origin other than blood accounts for circulating *miR-139-5p* in patients who suffered an AMI. In this respect, Ming et al. have reported decreased levels of *miR-139-5p* in left ventricle tissue of patients with hypertrophic cardiomyopathy and demonstrated an anti-hypertrophic effect mediated by this miRNA through the regulation of IGF-R1 and c-Jun [35]. It is known that IFG-R1 is a component of both MAPK and PI3K-Akt signalling pathways [36]. Interestingly, the assessment of miRNAs in-cluster individual weight in our study evidenced the presence of *miR-139-5p* and *miR-107* in all clusters. Further, the in silico analysis revealed that both miRNAs were implicated in the MAPK and PI3K-AKT signalling pathways. Voellenkle et al. also detected *miR-107* down-regulation in cHF compared to their controls [34]. Some reports have associated this miRNA with metabolism and cardiac function [37], as well as angiogenesis [38] or hypoxia [39]. Further, some reports indicate that *miR-107* regulates gene expression through the PI3K-AKT and MAPK pathways [40,41], in correlation with our in silico results. Moreover, *miR-30b-5p* was found to be deregulated in HF compared to both, healthy subjects and patients with shortness of breath [30].

To our knowledge, this is the first time *miR-335-5p* has been identified in patients recruited in a heart failure clinic, that is, patients with clinical evidence and diagnosis of chronic heart failure. Some studies including ischaemic patients detected a down-regulation of this miRNA in acute ischaemia [6] or during a three-month post-myocardial infarction period [42]. The latter study suggested *miR-335-5p* together with five other miRNAs as potential markers of deleterious left ventricular remodelling, but it did not provide evidence of changes in patients with established chronic heart failure. Studies in animal models have also related this miRNA with atherosclerosis [43] or ventricular remodelling [42,44,45], with apparently controversial findings. Thus, studies in mice have associated *miR-335-5p* overexpression with a reduction of atherosclerotic plaque vulnerability and angiogenesis promotion [46]. In contrast, this miRNA was found up-regulated in rat and mouse models of pulmonary hypertension, where the induced right ventricular remodelling was linked to increased *miR-335-5p* expression and its blockade alleviated remodelling [45]. Interestingly, contrasting results were observed in a model of sepsis-induced myocardial injury [44]. Long and colleagues observed that up-regulated levels of this miRNA ameliorated myocardial injury by reducing apoptosis and Ca^2+^ overload [44]. Nevertheless, we need to take into consideration that miRNA patterns in animal models may not reflect a human profile [47] and that all those studies reflect an acute injury rather than a chronic disease. Similarly, little is known about *miR-342-3p*. To this respect, Ellis et al. observed down-regulated levels of *miR-342-3p* in cHF in comparison to healthy subjects or patients with shortness of breath [30], but no association of this miRNA with background aetiology or ejection fraction in cHF has been reported.

The in silico analysis we performed with the validated miRNAs (main PPI network) revealed an enrichment of several pathways, including cell cycle, cellular senescence, and Ras, chemokine, PI3K-AKT and TGF-β signalling pathways. Multiple pathophysiological mechanisms are involved in HF. Among them, cHF patients are characterised by presenting an unresolving inflammatory state, endothelial dysfunction and cardiac remodelling (including cardiac fibrosis, hypertrophy and apoptosis). In agreement with our results, Ras/MAPK and PI3K-AKT signalling have been previously related to cardiac hypertrophy and HF [36,48]. Similarly, the TGF-β and PI3K-AKT pathways have been related to cardiac fibrosis [49,50,51]. Further, HF patients are characterised for presenting an unresolved inflammatory state, where chemokine levels are increased [52] and innate immunity cell increase their release of extracellular vesicles [53].

It is widely accepted that in HF there is a change in cardiac metabolism. In the healthy heart, cardiomyocytes produce energy mainly through fatty acid oxidation. However, in the failing heart, energy production swifts to a glucose-based metabolism (glycolysis and anaplerosis) and other forms of metabolism, such as the use of lactate, branched-chain amino acids and ketone bodies [54]. Although by in silico analysis we evidenced that miRNA-target genes in the DiffmiR cluster were associated with metabolism (glucagon signalling pathway, lipolysis regulation and carbohydrate digestion and absorption), a specific implication of these metabolic pathways in metabolic remodelling in cHF is, as far as we know, still undetermined. However, alterations in the glucagon signalling pathway (compromising cAMP signalling in cardiomyocytes) and increased levels of adipocyte lipolysis, have been detected in HF [55,56]. Further, suppression of HF development was observed in a mice model fed with a strict carbohydrate-restricted diet [57].

## 4. Materials and Methods

### 4.1. Study Design

Patients with cHF treated by guideline-directed medical therapy (GDMT) were prospectively recruited in the outpatient HF unit of the Hospital de la Santa Creu i Sant Pau (Barcelona, Spain) from September 2016 to July 2018 (*n* = 58). Patients with LVEF between 40% and 50% (mildly reduced LVEF; HFmrEF), past history of cancer, inflammatory disorders, sepsis or infection were excluded from the study. Pregnant women were also excluded. A group of reference subjects of age- and sex-matched subjects without cHF (*n* = 26) was also analysed.

A quantitative Low-density TaqMan™ Advanced miRNA Human A Card was run on 28 patients, 14 patients with preserved LVEF (HFpEF; LVFE ≥ 50%), and 14 with reduced LVEF (HFrEF; LVFE < 40%) (Table 4).

**Figure 9:**
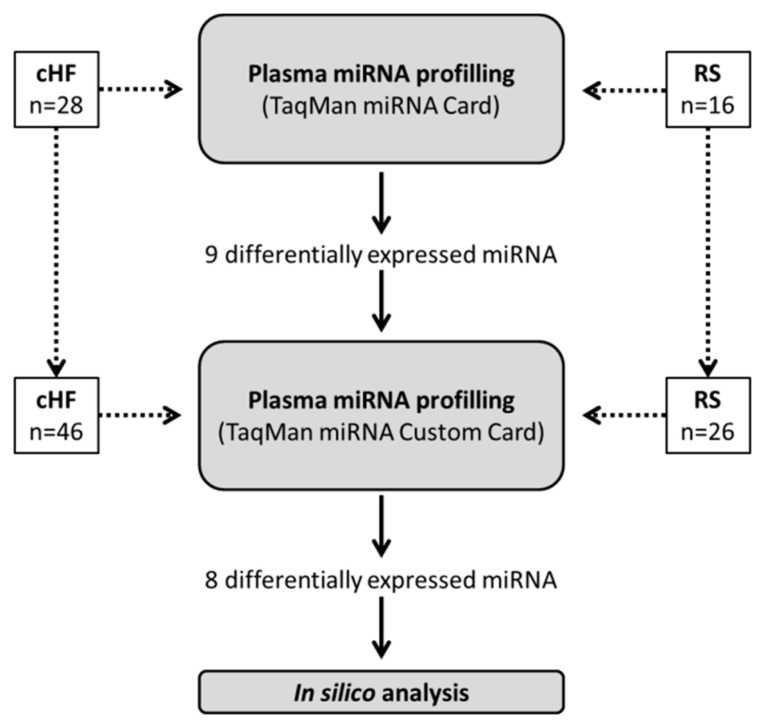
Schematic diagram of the study design. cHF: chronic heart failure; RS: reference subjects.

The reference-subject group comprised 16 sex- and age-matched subjects without cHF. Baseline demographic and biochemical data, classical CV risk factors, background medication and clinical outcomes of cHF patients and reference subjects are included in Table 4 and Table 5. A schematic diagram of the study design is shown in Figure 9.

The identified differential miRNAs were validated using the quantitative Low-density Custom TaqMan^®^ Array Advanced MicroRNA Cards in cHF patients (*n* = 58). Patients consistently expressing miRNA species in plasma were further investigated (*n* = 46). Samples of the reference subjects were similarly processed (*n* = 26). HFrEF affected 52.2% of patients, and 30.4% of cHF had ischaemic disease as the underlying cause of cHF. Clinical and biochemical characteristics, as well as clinical outcomes of these groups, are shown in Table 6 and Table 7.

### 4.2. Blood Sampling

After 10–14 h of fasting, venous blood was withdrawn without tourniquet into 3.8% sodium citrate tubes. All samples were processed identically and within the first 2 h. Firstly, blood was centrifuged at 1560× *g* for 20 min at room temperature to eliminate blood cells and generate platelet-poor plasma (PPP). Secondly, to obtain platelet-free plasma (PFP), samples were centrifuged at 1500× *g* for 10 min at room temperature. Finally, aliquots were stored at −80 °C until miRNA analyses were performed.

### 4.3. miRNA Extraction, cDNA Synthesis and Analysis

The isolation of miRNA from PFP was performed using the miRNeasy Serum/Plasma Advanced kit (217201; Qiagen; Hilden, Germany) following the provider’s instructions. During miRNA extraction, and according to the manufacturer’s directions, all samples were spiked-in with 25 fmol of the exogenous miRNA *cel-miR-39-3p*.

miRNAs were transcribed and pre-amplified using the TaqMan™ Advanced miRNA cDNA Synthesis Kit (A28007; Applied Biosystems; Foster City, CA, USA), and then profiled. For the initial miRNA identification and selection studies, miRNAs were profiled with the Low-density TaqMan™ Advanced miRNA Human A Card (A34714; Life Technologies; Carlsbad, CA, USA). This array focuses on highly characterised miRNAs [58]. Specifically, it contains 377 different human miRNAs, including 1 endogenous control (*hsa-miR-16-5p*). Additionally, it also comprises 1 exogenous control (*cel-miR-39-3p*) and 1 negative control (*ath-miR-159a*) that does not amplify in human samples. Validated miRNAs were profiled using the Low-density Custom TaqMan^®^ Array Advanced MicroRNA Cards (Life Technologies; Carlsbad, CA, USA) (Supplemental Table S8). Taqman RT-qPCR amplification was performed in an ABI PRISM 7900HT Sequence Detection System (Applied Biosystems; Foster City, CA, USA) and the amplification curves were analysed using the Thermo Fisher Connect Dashboard (Life Technologies; Carlsbad, CA, USA). Only miRNAs with an expression threshold below 35 Ct cycles were considered. Ct values were obtained and processed by the standard curve method and normalised with the exogenous miRNA *cel-miR-39-3p*, according to the equation 2^−(Ct [target]−Ct [exogenous])^.

### 4.4. In Silico Systems Biology Analysis

Putative target genes of the selected miRNAs were determined using the databases TargetScan, miRmap, miRWalk and miRDB [18,19,20,21,22]. Considered target genes were those with a high likelihood to be regulated by miRNA according to their search algorithms. For each miRNA, only those targets predicted in all four databases were selected for gene-network analysis.

Initial enrichment pathway analysis of curated target lists of the identified miRNAs was pursued with the web-based software ShinyGO [23]. The top-10 Gene Ontology terms, sorted by average and rank (FDR and fold), were selected.

Additionally, the curated target lists were imported into Cytoscape 3.0 (online free available platform) [25] to build protein-protein interaction (PPI) networks according to the STRING database interaction data [24]. A confidence cut-off was set to 0.8, and the maximum number of additional interactions was limited to 30. In order to identify PPI-enriched clusters, a community cluster strategy (GLay) [26] was applied to the main connected component of each network. The resulting clusters were then analysed for functional enrichments according to the pathway annotations from the KEGG database [27,28].

For individual miRNA pathway analysis, a repository-based gene database (RBGD) was created by querying “heart failure” to the STRING Pubmed database interaction data (bioinformatics tool of the Cytoscape platform). Predicted targets of selected miRNAs were then matched to the RBGD list. Common genes and their neighbour interacting genes were imported to ShinyGO for pathway enrichment analysis. A schematic diagram of the protocol used can be found in Supplemental Figure S1.

### 4.5. Statistical Analysis

Sample normality was assessed with the Shapiro–Wilks test. Qualitative variables were described using the number of cases and percentages and analysed with the Chi-squared test, while quantitative variables were described using the median [interquartile range] and analysed with the U Mann–Whitney test. Receiver Operating Characteristic (ROC) curve analyses and the corresponding area under the curve (AUC), along with its 95% confidence interval (CI) were calculated to determine the discrimination potential of miRNAs (patients vs. reference subjects), and AUCs values (with cut-offs ≥0.7 and ≤0.3) were used for miRNA selection. Additionally, ROC curves for predicted probabilities were pursued to identify expression thresholds of miRNAs between patients and reference subjects, between cHF types (HFpEF and HFrEF), background aetiology (ischaemic and non-ischaemic), or need for rehospitalisation during the 4–6 years of follow-up, and the corresponding area under the AUC with its 95% CI was calculated. A *p <* 0.05 was considered statistically significant. SPSS Statistical Analysis System (version 26.0, IBM Corp; Armonk, NY, USA) was utilised.

## 5. Conclusions

In conclusion, in this hypothesis-generating study, we have identified and validated a differential pattern of eight miRNAs that are down-regulated in the systemic circulation of cHF patients. Additionally, we have shown by in silico analysis and systems biology approaches that these miRNAs are, when considered as a cluster, involved in molecular mechanisms and signalling pathways related to cHF. Nevertheless, further research on this topic, including pursuing mechanistic studies to validate our results, is warranted to corroborate these findings as well as to improve the understanding of cHF pathophysiology.

### Study Limitations

This study is not free of limitations. Comparisons were pursued between cHF and a reference group of healthy subjects. This latter group was used to determine baseline miRNA levels for comparison purposes, as there are no established “normal” miRNA levels. Further studies to identify the effect of miRNAs in other populations are warranted.

## Figures and Tables

**Table 1:** Pathway enrichment analysis of GO terms in ShinyGo, sorted by average ranks (FDR and fold).

GO TERMS	Gene Number (Pathway Gene Number)	Fold Enrichment	Enrichment FDR
**Biological Process**			
Presynapse assembly	11 (52)	8.3	1.02 × 10^−5^
Coronary vasculature morphogenesis	6 (16)	14.8	1.79 × 10^−4^
Presynapse organisation	11 (55)	7.9	1.83 × 10^−5^
Coronary vasculature development	9 (46)	7.7	1.95 × 10^−4^
Synaptic membrane adhesion	7 (28)	9.8	3.69 × 10^−4^
Positive regulation of TGF-β signalling pathway	7 (30)	9.2	5.32 × 10^−4^
Positive regulation of cellular response to TGF-β stimulus	7 (30)	9.2	5.32 × 10^−4^
Ventricular septum development	11(72)	6.0	1.92 × 10^−4^
Cardiac septum development	14 (108)	5.1	7.89 × 10^−5^
Ventricular compact myocardium morhogenesis	4 (8)	19.7	1.23 × 10^−3^
**Molecular Function**			
SMAD binding	14 (80)	6.9	2.44 × 10^−6^
RNA strand annealing activity	3 (5)	23.7	5.l0 × 10^−3^
Insulin-like growth factor I binding	4 (13)	12.1	7.17 × 10^−3^
β-catenin binding	13 (89)	5.7	2.58 × 10^−5^
RNA stem-loop binding	4 (14)	11.3	9.53 × 10^−3^
Poly-purine tract binding	6 (29)	8.1	2.71 × 10^−3^
Transcription factor binding	42 (596)	2.7	2.36 × 10^−6^
Poly(G) binding	3 (8)	14.8	1.80 × 10^−2^
DNA-binding transcription repressor activity. RNA polymerase II-specific	21 (333)	2.4	4.50 × 10^−3^
3,5-cyclic-AMP phosphodiesterase activity	4 (16)	9.8	1.52 × 10^−2^
**Cellular Component**			
Cytoplasmic ribonucleoprotein granule	23 (272)	3.3	5.56 × 10^−5^
GABA-ergic synapse	9 (74)	4.8	4.56 × 10^−3^
Ribonucleoprotein granule	23 (288)	3.1	1.29 × 10^−4^
Node of Ranvier	4 (15)	10.5	1.l0 × 10^−2^
Filopodium	11 (119)	3.6	7.40 × 10^−3^
Glutamatergic synapse	25 (358)	2.7	4.44 × 10^−4^
Site of polarised growth	15 (196)	3.0	6.17 × 10^−3^
Chromatin	76 (1316)	2.2	8.70 × 10^−9^
Adherens junction	14 (180)	3.0	7.33 × 10^−3^
Heterochromatin	8 (77)	4.1	1.62 × 10^−2^

**Table 2:** ROC curve analysis of miRNAs according to their cluster formation.

	AUC ± SD (95% CI)	*p*-Value	Sensitivity	Specificity
**(a) Differentiation of Ejection Fraction**				
Clusters 1–4 miRNAs *	0.725 ± 0.102 (0.526–0.925)	**0.047**	0.769	0.643
Cluster 5 and 7 miRNAs: *miR-107 + miR-139-5p + miR-150-5p*	0.782 ± 0.083 (0.618–0.945)	**0.007**	0.722	0.714
Cluster 6 miRNAs: *miR-107 + miR-139-5p + let-7a-5p*	0.582 ± 0.112 (0.363–0.802)	0.467	0.571	0.538
Cluster 8 miRNAs: *miR-107 + miR-139-5p + miR-342-3p*	0.690 ± 0.097 (0.500–0.881)	0.068	0.611	0.643
**(b) Differentiation of Underlying Aetiology**				
Cluster 1–4 miRNAs *	0.789 ± 0.088 (0.617–0.962)	**0.019**	0.737	0.750
Cluster 5 and 7 miRNAs: *miR-107 + miR-139-5p + miR-150-5p*	0.643 ± 0.l18 (0.410–0.875)	0.216	0.696	0.667
Cluster 6 miRNAs: *miR-107 + miR-139-5p + let-7a-5p*	0.691 ± 0.126 (0.443–0.939)	0.124	0.684	0.625
Cluster 8 miRNAs: *miR-107 + miR-139-5p + miR-342-3p*	0.645 ± 0.106 (0.438–0.853)	0.193	0.636	0.500
**(c) Differentiation of Need for Rehospitalisation**				
Clusters 1–4 miRNAs *	0.518 ± 0.118 (0.287–0.749)	0.880	0.471	0.600
Cluster 5 and 7 miRNAs: *miR-107 + miR-139-5p + miR-150-5p*	0.695 ± 0.092 (0.514–0.877)	0.080	0.636	0.600
Cluster 6 miRNAs: *miR-107 + miR-139-5p + let-7a-5p*	0.641 ± 0.107 (0.432–0.850)	0.228	0.588	0.600
Cluster 8 miRNAs: *miR-107 + miR-139-5p + miR-342-3p*	0.700 ± 0.093 (0.518–0.882)	0.074	0.591	0.600

* Includes all miRNAs: *let-7a-5p*, *miR-107*, *miR-125a-5p*, *miR-139-5p*, *miR-150-5p*, *miR-30b-5p* and *miR-342-3p*. Numbers in bold indicate statistical significance. (a) Ejection fraction: HFrEF (LVEF < 40; *n* = 24) and HFpEF (LVEF ≥ 50; *n* = 22); (b) Background aetiology: ischaemic (*n* = 14) and non-ischaemic (*n* = 32); (c) Need for rehospitalisation: rehospitalised (*n* = 32) and non-rehospitalised (*n* = 14). AUC: area under the curve; CI: confidence interval; HFpEF: heart failure with preserved ejection fraction; HFrEF: heart failure with reduced ejection fraction; LVEF: left ventricular ejection fraction; ROC: receiver operating characteristic; SD: standard deviation.

**Table 3:** Clustered miRNA targets detected in the STRING-generated heart failure database.

miRNA	Target	Cluster
*miR-107*	ESR1	1
*miR-107*	BDNF	1
*miR-107*	MFN2	4
*miR-139-5p*	DMD	1
*miR-139-5p*	ROCK2	1
*miR-139-5p*	MAPK8	1
*miR-139-5p*	GNB1	1
*miR-139-5p*	ROCK1	1
*miR-139-5p*	IGF1R	1
*miR-139-5p*	SMARCA4	2
*miR-139-5p*	NOTCH1	2
*miR-139-5p*	PDE3A	12

**Table 4:** Baseline characteristics of cHF patients and reference subjects in the miRNA selection.

	cHF *n* = 28	Reference Subjects *n* = 16	*p*-Value
**Demographic Characteristics; mean ± SD**			
Male/Female, *n*	14/14	10/6	0.423
Age, years	69 ± 12	67 ± 8	0.222
Systolic blood pressure, mmHg	119 ± 20	140 ± 17	0.001
Diastolic blood pressure, mmHg	75 ± 11	79± 11	0.191
Left ventricular ejection fraction,%	43 ± 20	52–74 *	-
**Clinical History; *n* (%)**			
**cHF aetiology**			
Ischaemic	7 (25)	-	-
Non-ischaemic	21 (75)	-	-
Hypertensive cardiomyopathy	7 (25)	-	-
Dilated cardiomyopathy	7 (25)	-	-
Heart valve disease	7 (25)	-	-
Hypertrophic cardiomyopathy	0 (0)	-	-
**Left ventricular ejection fraction**			
HFpEF	14 (50)	-	-
HFrEF	14 (50)	-	-
**New York Heart Association cHF stage**			
NYHA I	0 (0)	-	-
NYHA II	11 (39.2)	-	-
NYHA III	17 (60.7)	-	-
NYHA IV	0 (0)	-	-
**Risk factors; *n*** (%)			
Smokers	0 (0)	0 (0)	-
Hypertension	18 (64.2)	10 (62.5)	0.906
Pulmonary hypertension	18 (64.2)		-
Diabetes mellitus	14 (50)	2 (12.5)	0.013
Dyslipidaemia	14 (50)	13 (81.2)	0.041
Chronic kidney disease	12 (42.8)	0 (0)	0.002
Atrial fibrillation	11 (39.2)		-
**Background medication; *n*** (%)			
Angiotensin-converting-enzyme inhibitors	13 (46.4)	8 (50)	0.820
Angiotensin II receptor blockers	10 (35.7)	3 (18.7)	0.235
Angiotensin receptor neprilysin inhibitors	4 (14.20)		-
Beta-blockers	24 (85.7)	1 (6.2)	0.000
Aldosterone antagonists	18 (64.2)	-	
Diuretics	26 (92.8)	1 (6.2)	0.000
Ivabradine	3 (10.7)	-	-
Statins	19 (67.8)	12 (75)	0.617
Insulin	4 (14.2)	1 (6.2)	0.419
Anti-diabetic drugs	10 (35.7)	2 (12.5)	0.096
Antiplatelet agents	7 (25)	5 (31.2)	0.654
Anticoagulants	16 (57.1)	0 (0)	0.000
Anti-arrhythmic drugs	6 (21.4)	-	-

cHF: chronic heart failure; HFpEF: heart failure with preserved ejection fraction; HFrEF: heart failure with reduced ejection fraction; NYHA: New York Heart Association; SD: standard deviation. Numbers in bold indicate statistical significance. * LVEF normal range (excerpted from https://www.ncbi.nlm.nih.gov/books/NBK459131/, on 18 January 2021).

**Table 5:** Biochemical and clinical outcomes of cHF patients in the miRNA selection.

	cHF *n* = 28
**Biochiemistry; mean ± SD**	
Haemoglobin, mg/dL	133 ± 19
Creatinine, mg/dL	1.24 ± 0.41
C-Reactive Protein, mg/mL	10.11 ± 12.99
NT-proBNP, pg/mL	3834 ± 4602
High-sensitive troponin T, ng/L	28 ± 17
Platelets, 10^3^/mm^3^	164 ± 47
Erythrocytes, 10^6^/mm^3^	4.1 ± 0.7
Leukocytes, mm^3^	7723 ± 2283
Osteopontin, ng/mL	99.06 ± 35.78
**Major outcomes during follow-up; *n* (%)**	
Cardiovascular event ^1^	12 (42.8)
Stroke	3 (10.7)
AMI	2 (7.1)
HTx	6 (21.4)
Cardiovascular death	5 (17.8)
Emergency hospital admission for cHF	9 (32.1)
Rehospitalisation for cHF	19 (67.8)
Aortic aneurism	0 (0)
Other death causes ^2^	8 (28.5)

AMI: acute myocardial infarction; cHF: chronic heart failure; HTx: heart transplantation; NT-proBNP: N-terminal prohormone of brain natriuretic peptide; SD: standard deviation. ^1.^ Includes patients that suffered a stroke, an AMI, a cardiovascular death (mainly due to cHF), or were admitted to the emergency department. It does not include patients that underwent HTx or patients that were rehospitalised. ^2.^ Includes patients that died due to a septic shock, a haemorrhage, or a non-successful HTx.

**Table 6:** Baseline characteristics of cHF patients and reference subjects in the miRNA validation.

	cHF *n* = 46	Reference Subjects *n* = 26	*p*-Value
**Demographic characteristics; mean ± SD**			
Male/Female, *n*	31/15	17/9	0.862
Age, years	69 ± 11	66 ± 8	0.128
Systolic blood pressure, mmHg	118 ± 19	140 ± 18	**0.000**
Diastolic blood pressure, mmHg	75 ± 10	83 ± 14	**0.009**
Left ventricular ejection fraction, %	43 ± 17	52–74 *	
**Clinical history; *n* (%)**			
**cHF aetiology**			
Ischaemic	14 (30.4)	-	-
Non-ischaemic	32 (69.6)	-	-
Hypertensive cardiomyopathy	9 (19.6)	-	-
Dilated cardiomyopathy	12 (26.1)	-	-
Heart valve disease	8 (17.4)	-	-
Hypertrophic cardiomyopathy	3 (6.5)	-	-
**Left ventricular ejection fraction**			
HFpEF	22 (47.8)	-	-
HFrEF	24 (52.2)	-	-
**New York Heart Association cHF stage**			
NYHA I	0 (0)	-	-
NYHA II	17 (36.9)	-	-
NYHA III	29 (63.1)	-	-
NYHA IV	0 (0)	-	-
**Risk factors; *n* (%)**			
Smokers	1 (2.1)	4 (15.3)	**0.034**
Hypertension	34 (73.9)	16 (61.5)	0.274
Pulmonary hypertension	23 (50)	-	-
Diabetes mellitus	24 (52.2)	4 (15.3)	**0.002**
Dyslipidaemia	25 (54.3)	17 (65.3)	0.264
Chronic kidney disease	18 (39.1)	0 (0)	**0.000**
Atrial fibrillation	24 (52.2)	-	-
**Background medication; *n* (%)**			
Angiotensin-converting-enzyme inhibitors	21 (45.6)	12 (46.1)	0.967
Angiotensin II receptor blockers	14 (30.4)	5 (19.2)	0.300
Angiotensin receptor neprilysin inhibitors	5 (10.8)	-	-
Beta-blockers	40 (86.9)	1 (3.8)	**0.000**
Aldosterone antagonists	28 (60.8)	-	-
Diuretics	43 (93.4)	2 (7.6)	**0.000**
Ivabradine	4 (8.6)	-	-
Statins	32 (69.6)	15 (57.6)	0.309
Insulin	6 (13)	1 (3.8)	0.206
Anti-diabetic drugs	14 (30.4)	3 (11.5)	0.070
Antiplatelet agents	13 (28.2)	5 (19.2)	0.395
Anticoagulants	29 (63.1)	1 (3.8)	**0.000**
Anti-arrhythmic drugs	10 (21.7)	-	-

cHF: chronic heart failure; HFpEF: heart failure with preserved ejection fraction; HFrEF: heart failure with reduced ejection fraction; NYHA: New York Heart Association; SD: standard deviation. Numbers in bold indicate statistical significance. * LVEF normal range (excerpted from https://www.ncbi.nlm.nih.gov/books/NBK459131*/,* on 18 January 2021).

**Table 7:** Biochemical and clinical outcomes of cHF patients in the miRNA validation.

	cHF *n* = 46
**Biochemistry; mean ± SD**	
Haemoglobin, mg/dL	132 ± 19
Creatinine, mg/dL	1.27 ± 0.44
C-Reactive Protein, mg/mL	9.78 ± 15.34
NT-proBNP, pg/mL	3278 ± 3931
High-sensitive troponin T, ng/L	28 ± 17
Platelets, 10^3^/mm^3^	156 ± 53
Erythrocytes, 10^6^/mm^3^	4 ± 0.8
Leukocytes, mm^3^	7588 ± 2064
Osteopontin, ng/mL	100.17± 41.72
**Major outcomes during follow-up; *n* (%)**	
Cardiovascular event ^1^	24 (52.2)
Stroke	7 (15.2)
AMI	2 (4.3)
HTx	7 (15.2)
Cardiovascular death	9 (19.6)
Emergency hospital Admission for cHF	16 (34.7)
Rehospitalisation for cHF	32 (69.6)
Aortic aneurism	1 (2.1)
Other death causes ^2^	11 (23.9)

AMI: acute myocardial infarction; cHF: chronic heart failure; HTx: heart transplantation; NT-proBNP: N-terminal prohormone of brain natriuretic peptide; SD: standard deviation. ^1.^ Includes patients that suffered a stroke, an AMI, a cardiovascular death (mainly due to cHF), or were admitted to the emergency department. It does not include patients that underwent HTx or patients that were rehospitalised. ^2.^ Includes patients that died due to a septic shock, a haemorrhage, or a non-successful HTx.

## Data Availability

The data that support the findings of this study are available from the corresponding author upon reasonable request.

## Supplementary Materials

The following supporting information can be downloaded at: https://www.mdpi.com/article/10.3390/ijms232315226/s1.
