# Supplementary material for: Systems Biology in Chronic Heart Failure—Identification of Potential miRNA Regulators
Source: Int J Mol Sci. 2022 Dec 3;23(23):15226. doi: 10.3390/ijms232315226 (PMC9740605; doi:10.3390/ijms232315226)
Supplement: Supplementary file 1 [file ijms-23-15226-s001.zip › ijms-1994036-supplementary.pdf]

SUPPLEMENTAL MATERIAL

Systems Biology in Chronic Heart Failure – Identification of Potential miRNAs Regulators

Vilella-Figuerola A[1,2], Gallinat A[1,2], Escate R[1,3], Mirabet S[3,4], Padró T[1,3,†] Badimon L[1,3,5,†*]

[1]Cardiovascular-Program-ICCC, IR-Hospital Santa Creu i Sant Pau, IIB-Sant Pau, Barcelona, Spain
[2]Biochemistry and Molecular Biology Department, Universitat Autònoma de Barcelona (UAB), Barcelona, Spain
[3]Centro de Investigación Biomédica en Red Cardiovascular, Instituto de Salud Carlos III, Madrid, Spain
[4]Heart Failure Group, Cardiology Department, Hospital Santa Creu i Sant Pau, Barcelona, Spain
[5]UAB-Chair Cardiovascular Research, Barcelona, Spain
[†]This authors contributed equally to this work.

***Correspondence**:
Prof. Lina Badimon
lbadimon@santpau.cat
Cardiovascular Program-ICCC
Research Institute Hospital de la Santa Creu i Sant Pau
Sant Antoni M[a] Claret 167, 08025 Barcelona, Spain
Phone: +34.935565882
Fax: +34.935565559

Keywords: miRNA, chronic heart failure, network, enrichment pathways, pathophysiology

SUPPLEMENTAL FIGURES

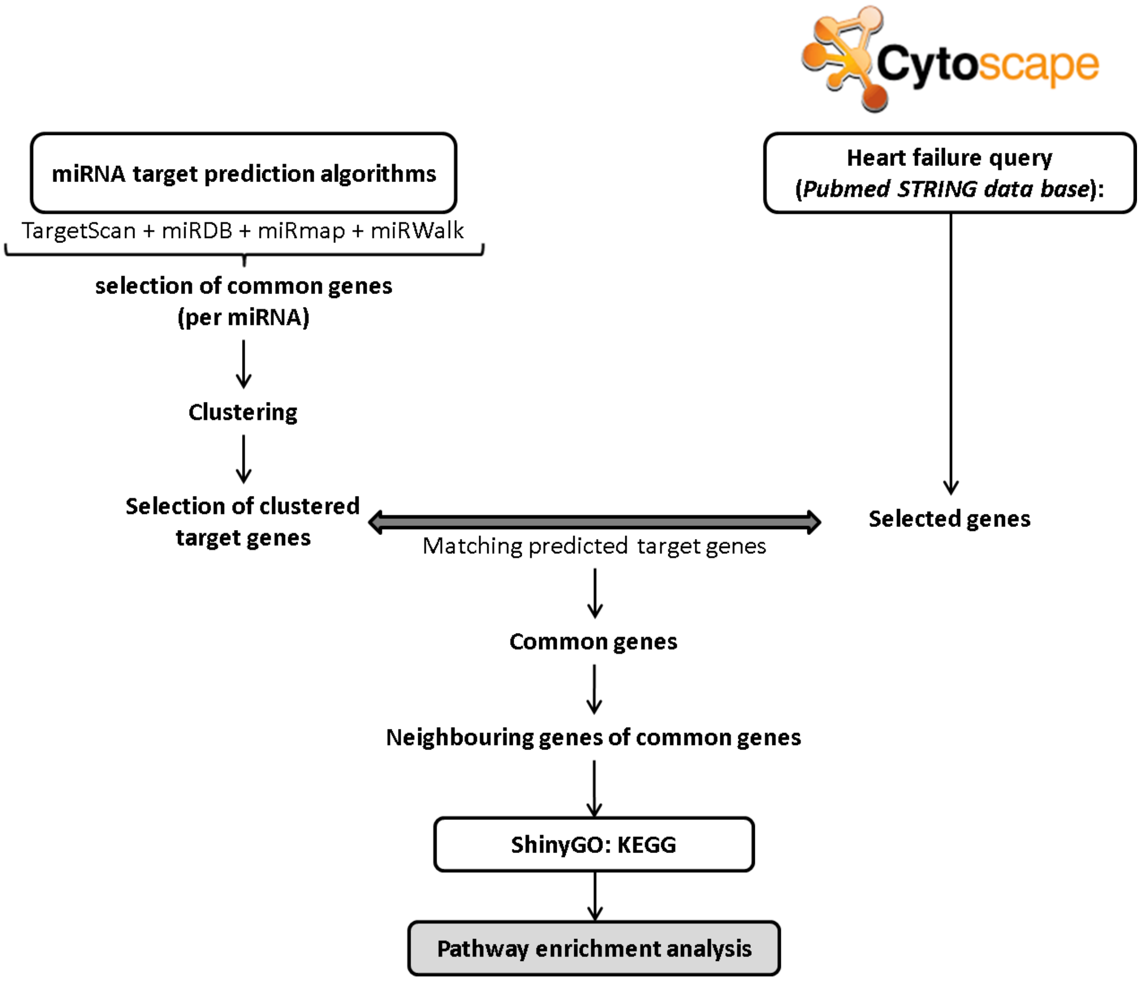

Supplemental Figure S1: Schematic representation of the *in silico* analysis of individual miRNAs.

SUPPLEMENTAL TABLES

Supplemental Table S1: miRNA selection strategy

	Fold-change (log_2)	AUC±SD (95% CI)	P-value	Same family	miRBase reads	Quartile reads
let-7a-5p	**−1.777**	**0.842±0.063 (0.718–0.966)**	**0.001**	**1**	**215,985,765**	**4**
miR-485-5p	−3.245	0.823±0.085 (0.657–0.989)	0.004	0	3,168	1
miR-342-3p	**−1.675**	**0.772±0.079 (0.617–0.928)**	**0.005**	**0**	**484,947**	**3**
miR-30b-5p	**−1.058**	**0.760±0.087 (0.590–0.931)**	**0.011**	**0**	**1,110,974**	**3**
miR-335-5p	**−0.995**	**0.749±0.086 (0.582–0.917)**	**0.012**	**0**	**416,898**	**3**
miR-125a-5p	**−0.957**	**0.748±0.080 (0.591–0.906)**	**0.012**	**0**	**1,146,942**	**3**
miR-150-5p	**−1.270**	**0.747±0.078 (0.594–0.900)**	**0.010**	**0**	**117,587**	**2**
miR-337-5p	−1.548	0.741±0.093 (0.558–0.923)	0.020	0	4,424	1
miR-224-5p	**−1.370**	**0.741±0.095 (0.555–0.926)**	**0.020**	**0**	**74,998**	**2**
miR-494-3p	−1.223	0.740±0.085 (0.574–0.907)	0.018	0	11,622	1
let-7g-5p	−0.791	0.739±0.082 (0.579–0.899)	0.016	1	50,508,337	4
let-7f-5p	−0.973	0.739±0.082 (0.579–0.899)	0.016	1	115,380,455	4
miR-107	**−0.968**	**0.737±0.090 (0.561–0.913)**	**0.018**	**0**	**5,491,348**	**4**
miR-495-3p	−0.894	0.730±0.087 (0.560–0.900)	0.021	0	20,270	1
miR-139-5p	**−1.299**	**0.729±0.088 (0.555–0.902)**	**0.028**	**0**	**85,265**	**2**
miR-301a-3p	−1.168	0.726±0.096 (0.539–0.914)	0.034	0	81,204	2
miR-26a-5p	−0.558	0.726±0.086 (0.557–0.895)	0.024	0	28,542,742	4
miR-328-3p	−0.740	0.722±0.097 (0.532–0.911)	0.032	0	26,824	1
miR-142-3p	−0.840	0.720±0.082 (0.559–0.881)	0.019	0	160,986	3
miR-377-3p	−1.001	0.701±0.087 (0.530–0.872)	0.044	0	4,424	1
miR-374b-5p	−0.593	0.701±0.088 (0.530–0.873)	0.044	0	491,420	3
miR-369-3p	−1.062	0.701±0.095 (0.514–0.887)	0.043	0	11,211	1
miR-574-3p	−0.763	0.700±0.092 (0.521–0.879)	0.050	0	125,985	3
miR-323a-3p	−0.659	0.697±0.091 (0.518–0.875)	0.049	0	603	1
miR-451a	0.421	0.286±0.080 (0.130–0.442)	0.021	0	1,666,621	4
miR-424-5p	0.726	0.269±0.080 (0.113–0.426)	0.016	0	861,807	3
miR-660-5p	0.591	0.263±0.085 (0.096–0.431)	0.017	0	91,879	2
miR-210-3p	1.137	0.253±0.091 (0.075–0.432)	0.023	0	66,359	2

AUC: area under the curve; **CI**: confidence interval; **SD**: standard deviation. **In bold**: selected miRNAs.
Selection strategy: 1) Selection of top 15 best miRNAs according to AUC and fold-change. 2) Elimination of miRNAs from the same family (miRNAs with better AUC selected). 3) Elimination of miRNAs with low number of reads (4th quantile).

Supplemental Table S2: Expression values of analysed miRNAs

miRNA	CTL (*n*=26) median [IQR]	cHF (*n*=46) median [IQR]	*p*-value	Fold-change (log_2)
let-7a-5p	0.00045 [0.00008–0.00158]	0.00012 [0.00003–0.0003]	**0.004**	0.266 (−1.911)
miR-107	0.00004 [0.00002–0.0001]	0.00002 [0.000003–0.00005]	**0.044**	0.620 (−0.689)
miR-125a-5p	0.00121 [0.00041–0.00167]	0.00031 [0.00012–0.00059]	**0.002**	0.252 (−1.987)
miR-139-5p	0.00006 [0.00002–0.00013]	0.00003 [0.00001–0.00005]	**0.006**	0.456 (−1.133)
miR-150-5p	0.00521 [0.00288–0.0088]	0.0021 [0.00083–0.00372]	**0.000**	0.403 (−1.312)
miR-30b-5p	0.00123 [0.00077–0.0042]	0.00053 [0.00032–0.00136]	**0.007**	0.430 (−1.217)
miR-335-5p	0.00165 [0.00021–0.00331]	0.00065 [0.00019–0.00107]	**0.048**	0.397 (−1.334)
miR-342-3p	0.0017 [0.00092–0.00338]	0.00045 [0.00013–0.00087]	**0.000**	0.264 (−1.921)
miR-224-5p	0.00006 [0.00002–0.00019]	0.00003 [0.00001–0.00007]	0.079	0.486 (−1.040)
miR-16-5p	0.00603 [0.0027–0.00943]	0.00511 [0.00249–0.00843]	0.694	0.847 (−0.240)
miR-223-3p	0.0243 [0.01047–0.03971]	0.01508 [0.00857–0.03479]	0.226	0.621 (−0.688)

cHF: chronic heart failure; **CTL**: controls; **IQR**: interquartile range
Bold indicates statistical significance.

Supplemental Table S3: Pathways identified by enrichment analysis included in Figure 3

	DESCRIPTION - KEGG	FDR value	Genes (background genes)	Fold enrichment
**CLUSTER 1**				
hsa04510	Focal adhesion	5.52×10^{-21}	21 (198)	10.61
hsa04062	Chemokine signalling pathway	3.89×10^{-20}	20 (186)	10.75
hsa04014	Ras signalling pathway	4.27×10^{-20}	21 (226)	9.29
hsa04151	PI3K-Akt signalling pathway	4.67×10^{-19}	23 (350)	6.57
hsa04917	Prolactin signalling pathway	1.60×10^{-17}	14 (69)	20.29
hsa01522	Endocrine resistance	2.05×10^{-17}	15 (95)	15.79
hsa04926	Relaxin signalling pathway	3.37×10^{-17}	16 (128)	12.5
hsa04915	Oestrogen signalling pathway	1.66×10^{-15}	15 (133)	11.28
hsa01521	EGFR tyrosine kinase inhibitor resistance	2.11×10^{-15}	13 (78)	16.67
hsa04012	ErbB signalling pathway	4.02×10^{-15}	13 (83)	15.66
hsa04935	Growth hormone synthesis, secretion and action	8.07×10^{-15}	14 (118)	11.86
hsa04015	Rap1 signalling pathway	1.82×10^{-14}	16 (202)	7.92
hsa04810	Regulation of actin cytoskeleton	2.82×10^{-14}	16 (209)	7.66
hsa04722	Neurotrophin signalling pathway	1.24×10^{-13}	13 (114)	11.4
hsa04912	GnRH signalling pathway	2.20×10^{-13}	12 (89)	13.48
hsa04933	AGE-RAGE signalling pathway in diabetic complications	5.44×10^{-13}	12 (98)	12.24
hsa04910	Insulin signalling pathway	6.05×10^{-13}	13 (133)	9.77
hsa04010	MAPK signalling pathway	1.77×10^{-12}	16 (288)	5.56
hsa04370	VEGF signalling pathway	2.44×10^{-12}	10 (57)	17.54
hsa04071	Sphingolipid signalling pathway	2.53×10^{-12}	12 (116)	10.34
hsa04919	Thyroid hormone signalling pathway	3.24×10^{-12}	12 (119)	10.08
hsa04611	Platelet activation	4.01×10^{-12}	12 (122)	9.84
hsa04068	FoxO signalling pathway	6.05×10^{-12}	12 (127)	9.45
hsa04520	Adherens junction	8.53×10^{-12}	10 (67)	14.93
hsa04660	T cell receptor signalling pathway	1.30×10^{-11}	11 (101)	10.89
hsa04550	Signalling pathways regulating pluripotency of stem cells	1.54×10^{-11}	12 (140)	8.57
hsa04921	Oxytocin signalling pathway	2.84×10^{-11}	12 (149)	8.05
hsa04664	Fc epsilon RI signalling pathway	1.92×10^{-10}	9 (66)	13.64
hsa04625	C-type lectin receptor signalling pathway	2.67×10^{-10}	10 (102)	9.8
hsa04072	Phospholipase D signalling pathway	3.70×10^{-10}	11 (147)	7.48
hsa04150	mTOR signalling pathway	4.77×10^{-10}	11 (151)	7.28
hsa04540	Gap junction	1.43×10^{-09}	9 (87)	10.34
hsa05418	Fluid shear stress and atherosclerosis	2.03×10^{-09}	10 (130)	7.69
hsa04371	Apelin signalling pathway	2.14×10^{-09}	10 (131)	7.63
hsa04929	GnRH secretion	3.09×10^{-09}	8 (63)	12.7
hsa04670	Leukocyte transendothelial migration	8.23×10^{-09}	9 (109)	8.26
hsa04662	B cell receptor signalling pathway	1.36×10^{-08}	8 (78)	10.26
hsa04720	Long-term potentiation	8.54×10^{-08}	7 (64)	10.94
hsa04024	cAMP signalling pathway	1.16×10^{-07}	10 (208)	4.81
hsa04650	Natural killer cell mediated cytotoxicity	2.93×10^{-07}	8 (121)	6.61
hsa04210	Apoptosis	5.46×10^{-07}	8 (132)	6.06
hsa04211	Longevity regulating pathway	5.58×10^{-07}	7 (87)	8.05
hsa04270	Vascular smooth muscle contraction	5.64×10^{-07}	8 (133)	6.02
hsa04530	Tight junction	1.76×10^{-06}	8 (156)	5.13
hsa04144	Endocytosis	4.14×10^{-06}	9 (241)	3.73
hsa04666	Fc gamma R-mediated phagocytosis	1.18×10^{-05}	6 (90)	6.67
hsa04723	Retrograde endocannabinoid signalling	1.32×10^{-05}	7 (145)	4.83
hsa04218	Cellular senescence	1.61×10^{-05}	7 (150)	4.67
hsa05142	Chagas disease	1.91×10^{-05}	6 (99)	6.06
hsa04928	Parathyroid hormone synthesis, secretion and action	2.34×10^{-05}	6 (103)	5.83
hsa04022	cGMP-PKG signalling pathway	2.53×10^{-05}	7 (162)	4.32
hsa04066	HIF-1 signalling pathway	2.69×10^{-05}	6 (106)	5.66
hsa04668	TNF signalling pathway	3.52×10^{-05}	6 (112)	5.36
hsa04960	Aldosterone-regulated sodium reabsorption	8.51×10^{-05}	4 (37)	10.81
hsa04261	Adrenergic signalling in cardiomyocytes	1.50×10^{-04}	6 (147)	4.08
hsa04657	IL-17 signalling pathway	1.80×10^{-04}	5 (92)	5.43
hsa04930	Type II diabetes mellitus	1.80×10^{-04}	4 (46)	8.7

	DESCRIPTION - KEGG	FDR value	Genes (background genes)	Fold enrichment
hsa04750	Inflammatory mediator regulation of TRP channels	$1.90x10^{-04}$	5 (94)	5.32
hsa04630	JAK-STAT signalling pathway	$2.20x10^{-04}$	6 (160)	3.75
hsa04620	Toll-like receptor signalling pathway	$2.60x10^{-04}$	5 (101)	4.95
hsa04659	Th17 cell differentiation	$2.60x10^{-04}$	5 (101)	4.95
hsa01524	Platinum drug resistance	$7.90x10^{-04}$	4 (70)	5.71
hsa04918	Thyroid hormone synthesis	$9.60x10^{-04}$	4 (74)	5.41
hsa05412	Arrhythmogenic right ventricular cardiomyopathy	0.001	4 (76)	5.26
hsa04310	Wnt signalling pathway	0.0016	5 (154)	3.25
hsa04350	TGF-β signalling pathway	0.0019	4 (91)	4.4
hsa05414	Dilated cardiomyopathy	0.0022	4 (95)	4.21
hsa04621	NOD-like receptor signalling pathway	0.0026	5 (174)	2.87
hsa04931	Insulin resistance	0.0034	4 (107)	3.74
hsa04961	Endocrine and other factor-regulated calcium reabsorption	0.0045	3 (53)	5.66
hsa04923	Regulation of lipolysis in adipocytes	0.0047	3 (54)	5.56
hsa04152	AMPK signalling pathway	0.0049	4 (120)	3.33
hsa05017	Spinocerebellar ataxia	0.0074	4 (135)	2.96
hsa04714	Thermogenesis	0.008	5 (229)	2.18
hsa04920	Adipocytokine signalling pathway	0.0088	3 (69)	4.35
hsa04141	Protein processing in endoplasmic reticulum	0.0142	4 (165)	2.42
hsa04658	Th1 and Th2 cell differentiation	0.0158	3 (87)	3.45
hsa04512	ECM-receptor interaction	0.0162	3 (88)	3.41
hsa05410	Hypertrophic cardiomyopathy	0.0166	3 (89)	3.37
hsa04922	Glucagon signalling pathway	0.0229	3 (101)	2.97
hsa04973	Carbohydrate digestion and absorption	0.0375	2 (44)	4.55
**CLUSTER 2**				
hsa04110	Cell cycle	$3.13x10_{-14}$	14 (120)	11.67
hsa04218	Cellular senescence	$7.40x10_{-11}$	12 (150)	8
hsa04350	TGF-β signalling pathway	$2.47x10_{-10}$	10 (91)	10.99
hsa04659	Th17 cell differentiation	$2.89x10_{-07}$	8 (101)	7.92
hsa04550	Signalling pathways regulating pluripotency of stem cells	$2.12x10^{-06}$	8 (140)	5.71
hsa01522	Endocrine resistance	$2.58x10^{-06}$	7 (95)	7.37
hsa04310	Wnt signalling pathway	$3.76x10^{-06}$	8 (154)	5.19
hsa04919	Thyroid hormone signalling pathway	$9.76x10^{-06}$	7 (119)	5.88
hsa04068	FoxO signalling pathway	$1.38x10^{-05}$	7 (127)	5.51
hsa04330	Notch signalling pathway	$3.56x10^{-05}$	5 (52)	9.62
hsa04390	Hippo signalling pathway	$3.56x10^{-05}$	7 (153)	4.58
hsa04933	AGE-RAGE signalling pathway in diabetic complications	$3.56x10^{-05}$	6 (98)	6.12
hsa04520	Adherens junction	$9.10x10^{-05}$	5 (67)	7.46
hsa04371	Apelin signalling pathway	0.0017	5 (131)	3.82
hsa04917	Prolactin signalling pathway	0.0017	4 (69)	5.8
hsa04115	p53 signalling pathway	0.0019	4 (72)	5.56
hsa04630	JAK-STAT signalling pathway	0.0036	5 (160)	3.13
hsa05142	Chagas disease	0.0052	4 (99)	4.04
hsa04926	Relaxin signalling pathway	0.0125	4 (128)	3.13
hsa05418	Fluid shear stress and atherosclerosis	0.0129	4 (130)	3.08
hsa04658	Th1 and Th2 cell differentiation	0.0347	3 (87)	3.45
hsa04657	IL-17 signalling pathway	0.0395	3 (92)	3.26
**CLUSTER 3**				
hsa03013	RNA transport	$9.30x10^{-05}$	7 (160)	4.38
**CLUSTER 4**				
hsa04120	Ubiquitin mediated proteolysis	$3.14x10^{-08}$	9 (135)	6.67
hsa04310	Wnt signalling pathway	$1.22x10^{-06}$	8 (154)	5.19
hsa04390	Hippo signalling pathway	0.00015	6 (153)	3.92
hsa04340	Hedgehog signalling pathway	0.0086	3 (47)	6.38
hsa04550	Signalling pathways regulating pluripotency of stem cells	0.0128	4 (140)	2.86

Supplemental Table S4: Pathways identified by enrichment analysis of miRNAs included in Figure 4

	DESCRIPTION - KEGG	FDR value	Genes (background genes)	Fold enrichment
**CLUSTER 1**				
hsa04014	Ras signalling pathway	$5.82x10^{-17}$	18 (226)	7.96
hsa04510	Focal adhesion	$1.31x10^{-16}$	17 (198)	8.59
hsa04062	Chemokine signalling pathway	$5.14x10^{-13}$	14 (186)	7.53
hsa04151	PI3K-Akt signalling pathway	$5.14x10^{-13}$	17 (350)	4.86
hsa04012	ErbB signalling pathway	$1.19x10^{-12}$	11 (83)	13.25
hsa04810	Regulation of actin cytoskeleton	$1.62x10^{-12}$	14 (209)	6.70
hsa04520	Adherens junction	$4.71x10^{-12}$	10 (67)	14.93
hsa01521	EGFR tyrosine kinase inhibitor resistance	$1.72x10^{-11}$	10 (78)	12.82
hsa04015	Rap1 signalling pathway	$1.72x10^{-11}$	13 (202)	6.44
hsa04722	Neurotrophin signalling pathway	$1.72x10^{-11}$	11 (114)	9.65
hsa01522	Endocrine resistance	$7.0x10^{-11}$	10 (95)	10.53
hsa04917	Prolactin signalling pathway	$1.31x10^{-10}$	9 (69)	13.04
hsa04010	MAPK signalling pathway	$6.06x10^{-10}$	13 (288)	4.51
hsa04370	VEGF signalling pathway	$9.97x10^{-10}$	8 (57)	14.04
hsa04625	C-type lectin receptor signalling pathway	$2.4x10^{-9}$	9 (102)	8.82
hsa04670	Leukocyte transendothelial migration	$3.89x10^{-9}$	9 (109)	8.26
hsa04071	Sphingolipid signalling pathway	$5.46x10^{-9}$	9 (116)	7.76
hsa04935	Growth hormone synthesis, secretion and action	$6.13x10^{-9}$	9 (118)	7.63
hsa04068	FoxO signalling pathway	$1.1x10^{-8}$	9 (127)	7.09
hsa04926	Relaxin signalling pathway	$1.14x10^{-8}$	9 (128)	7.03
hsa05418	Fluid shear stress and atherosclerosis	$1.27x10^{-8}$	9 (130)	6.92
hsa04910	Insulin signalling pathway	$1.5x10^{-8}$	9 (133)	6.77
hsa04912	GnRH signalling pathway	$1.5x10^{-8}$	8 (89)	8.99
hsa04915	Oestrogen signalling pathway	$1.5x10^{-8}$	9 (133)	6.77
hsa04550	Signalling pathways regulating pluripotency of stem cells	$2.14x10^{-8}$	9 (140)	6.43
hsa04933	AGE-RAGE signalling pathway in diabetic complications	$2.88x10^{-8}$	8 (98)	8.16
hsa04072	Phospholipase D signalling pathway	$3.07x10^{-8}$	9 (147)	6.12
hsa04921	Oxytocin signalling pathway	$3.37x10^{-8}$	9 (149)	6.04
hsa04660	T cell receptor signalling pathway	$3.38x10^{-8}$	8 (101)	7.92
hsa04919	Thyroid hormone signalling pathway	$1.03x10^{-7}$	8 (119)	6.72
hsa04611	Platelet activation	$1.21x10^{-7}$	8 (122)	6.56
hsa04150	mTOR signalling pathway	$5.35x10^{-7}$	8 (151)	5.30
hsa04664	Fc epsilon RI signalling pathway	$1.16x10^{-6}$	6 (66)	9.09
hsa04144	Endocytosis	$1.32x10^{-6}$	9 (241)	3.73
hsa04024	cAMP signalling pathway	$4.82x10^{-6}$	8 (208)	3.85
hsa04530	Tight junction	$8.49x10^{-6}$	7 (156)	4.49
hsa04929	GnRH secretion	$1.93x10^{-5}$	5 (63)	7.94
hsa04650	Natural killer cell mediated cytotoxicity	$2.62x10^{-5}$	6 (121)	4.96
hsa04371	Apelin signalling pathway	$3.94x10^{-5}$	6 (131)	4.58
hsa04210	Apoptosis	$4.06x10^{-5}$	6 (132)	4.55
hsa04270	Vascular smooth muscle contraction	$4.18x10^{-5}$	6 (133)	4.51
hsa04662	B cell receptor signalling pathway	$4.72x10^{-5}$	5 (78)	6.41
hsa04540	Gap junction	$7.7x10^{-5}$	5 (87)	5.75
hsa04218	Cellular senescence	$7.78x10^{-5}$	6 (150)	4.00
hsa04666	Fc gamma R-mediated phagocytosis	$8.79x10^{-5}$	5 (90)	5.56
hsa04657	IL-17 signalling pathway	$9.61x10^{-5}$	5 (92)	5.43
hsa04022	cGMP-PKG signalling pathway	$1.1x10^{-4}$	6 (162)	3.70
hsa04659	Th17 cell differentiation	$1.4x10^{-4}$	5 (101)	4.95
hsa04066	HIF-1 signalling pathway	$1.7x10^{-4}$	5 (106)	4.72
hsa04668	TNF signalling pathway	$2.2x10^{-4}$	5 (112)	4.46
hsa04720	Long-term potentiation	$3.4x10^{-4}$	4 (64)	6.25
hsa04920	Adipocytokine signalling pathway	$4.5x10^{-4}$	4 (69)	5.80
hsa05412	Arrhythmogenic right ventricular cardiomyopathy	$6.2x10^{-4}$	4 (76)	5.26
hsa04211	Longevity regulating pathway	0.001	4 (87)	4.60
hsa04350	TGF-β signalling pathway	0.0012	4 (91)	4.40
hsa04960	Aldosterone-regulated sodium reabsorption	0.0012	3 (37)	8.11
hsa04621	NOD-like receptor signalling pathway	0.0014	5 (174)	2.87

	DESCRIPTION - KEGG	FDR value	Genes (background genes)	Fold enrichment
hsa05142	Chagas disease	0.0015	4 (99)	4.04
hsa04620	Toll-like receptor signalling pathway	0.0016	4 (101)	3.96
hsa04928	Parathyroid hormone synthesis, secretion and action	0.0018	4 (103)	3.88
hsa04931	Insulin resistance	0.002	4 (107)	3.74
hsa04930	Type II diabetes mellitus	0.0021	3 (46)	6.52
hsa04723	Retrograde endocannabinoid signalling	0.0058	4 (145)	2.76
hsa04261	Adrenergic signalling in cardiomyocytes	0.006	4 (147)	2.72
hsa01524	Platinum drug resistance	0.0065	3 (70)	4.29
hsa04310	Wnt signalling pathway	0.0069	4 (154)	2.60
hsa04630	JAK-STAT signalling pathway	0.0078	4 (160)	2.50
hsa04141	Protein processing in endoplasmic reticulum	0.0087	4 (165)	2.42
hsa04658	Th1 and Th2 cell differentiation	0.0112	3 (87)	3.45
hsa05410	Hypertrophic cardiomyopathy	0.0118	3 (89)	3.37
hsa05414	Dilated cardiomyopathy	0.0137	3 (95)	3.16
hsa04110	Cell cycle	0.025	3 (120)	2.50
hsa04152	AMPK signalling pathway	0.025	3 (120)	2.50
hsa04217	Necroptosis	0.0428	3 (149)	2.01
hsa05416	Viral myocarditis	0.0439	2 (55)	3.64
hsa04390	Hippo signalling pathway	0.0451	3 (153)	1.96
**CLUSTER 2**				
hsa04330	Notch signalling pathway	$1.44x10^{-6}$	6 (52)	11.54
hsa04350	TGF-β signalling pathway	$1.44x10^{-6}$	7 (91)	7.69
hsa04110	Cell cycle	$5.51x10^{-6}$	7 (120)	5.83
hsa04919	Thyroid hormone signalling pathway	$5.51x10^{-6}$	7 (119)	5.88
hsa04218	Cellular senescence	$2.2x10^{-4}$	6 (150)	4.00
hsa04310	Wnt signalling pathway	$2.4x10^{-4}$	6 (154)	3.90
hsa01522	Endocrine resistance	$3.2x10^{-4}$	5 (95)	5.26
hsa04659	Th17 cell differentiation	$3.7x10^{-4}$	5 (101)	4.95
hsa04520	Adherens junction	0.0012	4 (67)	5.97
hsa04550	Signalling pathways regulating pluripotency of stem cells	0.0013	5 (140)	3.57
hsa04068	FoxO signalling pathway	0.0095	4 (127)	3.15
hsa05418	Fluid shear stress and atherosclerosis	0.0099	4 (130)	3.08
hsa04917	Prolactin signalling pathway	0.0159	3 (69)	4.35
hsa04658	Th1 and Th2 cell differentiation	0.0273	3 (87)	3.45
hsa04933	AGE-RAGE signalling pathway in diabetic complications	0.0355	3 (98)	3.06
hsa04024	cAMP signalling pathway	0.0374	4 (208)	1.92
hsa04066	HIF-1 signalling pathway	0.0415	3 (106)	2.83
**CLUSTER 3**				
hsa04120	Ubiquitin mediated proteolysis	$2.34x10^{-12}$	11 (135)	8.15
hsa04310	Wnt signalling pathway	$6.6x10^{-6}$	7 (154)	4.55
hsa04390	Hippo signalling pathway	$6.18x10^{-5}$	6 (153)	3.92
hsa04340	Hedgehog signalling pathway	0.0057	3 (47)	6.38
hsa04550	Signalling pathways regulating pluripotency of stem cells	0.007	4 (140)	2.86
**CLUSTER 4**				
hsa03013	RNA transport	$8.56x10^{-5}$	6 (160)	3.75
hsa03040	Spliceosome	0.0115	4 (132)	3.03
**CLUSTER 5**				
hsa04742	Taste transduction	$1.38x10^{-5}$	4 (81)	4.94

Supplemental Table S5: Pathways identified by enrichment analysis of miRNAs included in Figure 5

	DESCRIPTION - KEGG	FDR value	Genes (background genes)	Fold enrichment
**CLUSTER 1**				
hsa04917	Prolactin signalling pathway	$1.02x10^{-13}$	9 (69)	13.04
hsa01522	Endocrine resistance	$4.11x10^{-11}$	8 (95)	8.42
hsa01521	EGFR tyrosine kinase inhibitor resistance	$5.55x10^{-10}$	7 (78)	8.97
hsa04151	PI3K-Akt signalling pathway	$5.55x10^{-10}$	10 (350)	2.86
hsa04012	ErbB signalling pathway	$6.43x10^{-10}$	7 (83)	8.43
hsa04630	JAK-STAT signalling pathway	$8.82x10^{-10}$	8 (160)	5.00
hsa04933	AGE-RAGE signalling pathway in diabetic complications	$1.55x10^{-9}$	7 (98)	7.14
hsa04062	Chemokine signalling pathway	$2.29x10^{-9}$	8 (186)	4.30
hsa04935	Growth hormone synthesis, secretion and action	$4.45x10^{-9}$	7 (118)	5.93
hsa04068	FoxO signalling pathway	$6.54x10^{-9}$	7 (127)	5.51
hsa04926	Relaxin signalling pathway	$6.54x10^{-9}$	7 (128)	5.47
hsa04915	Oestrogen signalling pathway	$7.42x10^{-9}$	7 (133)	5.26
hsa04659	Th17 cell differentiation	$6.3x10^{-8}$	6 (101)	5.94
hsa04625	C-type lectin receptor signalling pathway	$6.35x10^{-8}$	6 (102)	5.88
hsa05418	Fluid shear stress and atherosclerosis	$1.98x10^{-7}$	6 (130)	4.62
hsa04210	Apoptosis	$2.08x10^{-7}$	6 (132)	4.55
hsa04550	Signalling pathways regulating pluripotency of stem cells	$2.82x10^{-7}$	6 (140)	4.29
hsa04912	GnRH signalling pathway	$9.96x10^{-7}$	5 (89)	5.62
hsa04510	Focal adhesion	$1.42x10^{-6}$	6 (198)	3.03
hsa05142	Chagas disease	$1.46x10^{-6}$	5 (99)	5.05
hsa04015	Rap1 signalling pathway	$1.52x10^{-6}$	6 (202)	2.97
hsa04660	T cell receptor signalling pathway	$1.53x10^{-6}$	5 (101)	4.95
hsa04066	HIF-1 signalling pathway	$1.89x10^{-6}$	5 (106)	4.72
hsa04722	Neurotrophin signalling pathway	$2.62x10^{-6}$	5 (114)	4.39
hsa04014	Ras signalling pathway	$2.65x10^{-6}$	6 (226)	2.65
hsa04071	Sphingolipid signalling pathway	$2.73x10^{-6}$	5 (116)	4.31
hsa04910	Insulin signalling pathway	$4.94x10^{-6}$	5 (133)	3.76
hsa04370	VEGF signalling pathway	$6.37x10^{-6}$	4 (57)	7.02
hsa04921	Oxytocin signalling pathway	$7.9x10^{-6}$	5 (149)	3.36
hsa04929	GnRH secretion	$8.67x10^{-6}$	4 (63)	6.35
hsa04664	Fc epsilon RI signalling pathway	$9.84x10^{-6}$	4 (66)	6.06
hsa04920	Adipocytokine signalling pathway	$1.11x10^{-5}$	4 (69)	5.80
hsa04662	B cell receptor signalling pathway	$1.72x10^{-5}$	4 (78)	5.13
hsa04211	Longevity regulating pathway	$2.56x10^{-5}$	4 (87)	4.60
hsa04657	IL-17 signalling pathway	$3.13x10^{-5}$	4 (92)	4.35
hsa04750	Inflammatory mediator regulation of TRP channels	$3.35x10^{-5}$	4 (94)	4.26
hsa04620	Toll-like receptor signalling pathway	$4.28x10^{-5}$	4 (101)	3.96
hsa04931	Insulin resistance	$5.18x10^{-5}$	4 (107)	3.74
hsa04668	TNF signalling pathway	$6.09x10^{-5}$	4 (112)	3.57
hsa04919	Thyroid hormone signalling pathway	$7.57x10^{-5}$	4 (119)	3.36
hsa04611	Platelet activation	$8.22x10^{-5}$	4 (122)	3.28
hsa04371	Apelin signalling pathway	$1.0x10^{-4}$	4 (131)	3.05
hsa04010	MAPK signalling pathway	$1.2x10^{-4}$	5 (288)	1.74
hsa04930	Type II diabetes mellitus	$1.3x10^{-4}$	3 (46)	6.52
hsa04150	mTOR signalling pathway	$1.6x10^{-4}$	4 (151)	2.65
hsa04217	Necroptosis	$1.6x10^{-4}$	4 (149)	2.68
hsa04218	Cellular senescence	$1.6x10^{-4}$	4 (150)	2.67
hsa01524	Platinum drug resistance	$3.9x10^{-4}$	3 (70)	4.29
hsa04024	cAMP signalling pathway	$5.0x10^{-4}$	4 (208)	1.92
hsa04540	Gap junction	$7.0x10^{-4}$	3 (87)	3.45
hsa04658	Th1 and Th2 cell differentiation	$7.0x10^{-4}$	3 (87)	3.45
hsa04152	AMPK signalling pathway	0.0017	3 (120)	2.50
hsa04723	Retrograde endocannabinoid signalling	0.0028	3 (145)	2.07
hsa04072	Phospholipase D signalling pathway	0.0029	3 (147)	2.04
hsa04530	Tight junction	0.0034	3 (156)	1.92
hsa04621	NOD-like receptor signalling pathway	0.0044	3 (174)	1.72
hsa04973	Carbohydrate digestion and absorption	0.0047	2 (44)	4.55

	DESCRIPTION - KEGG	FDR value	Genes (background genes)	Fold enrichment
hsa04923	Regulation of lipolysis in adipocytes	0.0068	2 (54)	3.70
hsa04810	Regulation of actin cytoskeleton	0.007	3 (209)	1.44
hsa04720	Long-term potentiation	0.0092	2 (64)	3.13
hsa04520	Adherens junction	0.0098	2 (67)	2.99
hsa04666	Fc gamma R-mediated phagocytosis	0.0165	2 (90)	2.22
hsa04922	Glucagon signalling pathway	0.0199	2 (101)	1.98
hsa04928	Parathyroid hormone synthesis, secretion and action	0.0205	2 (103)	1.94
hsa04650	Natural killer cell mediated cytotoxicity	0.0274	2 (121)	1.65
hsa04261	Adrenergic signalling in cardiomyocytes	0.0391	2 (147)	1.36
hsa04310	Wnt signalling pathway	0.042	2 (154)	1.30
hsa04022	cGMP-PKG signalling pathway	0.0458	2 (162)	1.23
hsa04141	Protein processing in endoplasmic reticulum	0.0471	2 (165)	1.21
**CLUSTER 2**				
hsa04218	Cellular senescence	1.85×10^{-5}	5 (150)	3.33
hsa04350	TGF-β signalling pathway	4.21×10^{-5}	4 (91)	4.40
hsa04144	Endocytosis	8.0×10^{-4}	4 (241)	1.66
hsa04659	Th17 cell differentiation	0.0013	3 (101)	2.97
hsa04933	AGE-RAGE signalling pathway in diabetic complications	0.0013	3 (98)	3.06
hsa05142	Chagas disease	0.0013	3 (99)	3.03
hsa04110	Cell cycle	0.0019	3 (120)	2.50
hsa04371	Apelin signalling pathway	0.0023	3 (131)	2.29
hsa04550	Signalling pathways regulating pluripotency of stem cells	0.0026	3 (140)	2.14
hsa04390	Hippo signalling pathway	0.0032	3 (153)	1.96
hsa04010	MAPK signalling pathway	0.0168	3 (288)	1.04
hsa04520	Adherens junction	0.0168	2 (67)	2.99
hsa04722	Neurotrophin signalling pathway	0.0423	2 (114)	1.75
hsa04926	Relaxin signalling pathway	0.0481	2 (128)	1.56

Supplemental Table S6: Expression values of analysed miRNAs according to underlying aetiology of cHF

miRNA	Ischaemic cHF (*n*=14) median [IQR]	Non-ischaemic cHF (*n*=32) median [IQR]	*p*-value	Fold-change (log_2)
let-7a-5p	0.0001 [0.00006–0.00019]	0.00012 [0.00001–0.00043]	0.821	0.847 (−0.239)
miR-107	0.00002 [0.000002–0.00007]	0.00002 [0.000004–0.00005]	0.808	0.804 (−0.315)
miR-125a-5p	0.00012 [0.00006–0.00037]	0.00032 [0.00015–0.00061]	0.084	0.354 (−1.497)
miR-139-5p	0.00005 [0.00004–0.00006]	0.00002 [0.00001–0.00004]	**0.022**	1.929 (0.948)
miR-150-5p	0.0026 [0.00098–0.00357]	0.00173 [0.00054–0.00407]	0.412	1.503 (0.588)
miR-30b-5p	0.00054 [0.00018–0.00121]	0.00053 [0.00035–0.00136]	0.473	1.016 (0.023)
miR-335-5p	0.00083 [0.00008–0.00156]	0.00064 [0.00023–0.00098]	0.741	1.289 (0.367)
miR-342-3p	0.00028 [0.00007–0.00059]	0.00046 [0.00022–0.0009]	0.169	0.611 (−0.710)

cHF: chronic heart failure; **IQR**: interquartile range
Bold indicates statistical significance.

Supplemental Table S7: Expression values of analysed miRNAs according to cHF ejection fraction

miRNA	HFpEF (*n*=22) median [IQR]	HFrEF (*n*=24) median [IQR]	*p*-value	Fold-change (log_2)
let-7a-5p	0.00014 [0.00002–0.0003]	0.00011 [0.00004–0.00024]	0.947	1.209 (0.274)
miR-107	0.00001 [0.000002–0.00004]	0.00003 [0.00001–0.00009]	0.185	0.492 (−1.023)
miR-125a-5p	0.00031 [0.00015–0.00048]	0.00028 [0.00011–0.00062]	0.952	1.086 (0.119)
miR-139-5p	0.00002 [0.000004–0.00005]	0.00004 [0.00001–0.00005]	0.264	0.624 (−0.681)
miR-150-5p	0.00151 [0.00068–0.00293]	0.00278 [0.00098–0.00407]	0.130	0.546 (−0.874)
miR-30b-5p	0.00036 [0.00021–0.00092]	0.00107 [0.00038–0.00156]	0.075	0.336 (−1.572)
miR-335-5p	0.00065 [0.00032–0.00099]	0.00059 [0.00006–0.00144]	0.726	1.102 (0.140)
miR-342-3p	0.0004 [0.00006–0.00087]	0.00047 [0.00013–0.0009]	0.601	0.849 (−0.236)

cHF: chronic heart failure; **HFpEF**: heart failure with preserved ejection fraction; **HFrEF**: heart failure with reduced ejection fraction; **IQR**: interquartile range

Supplemental Table S8: Selected miRNAs

microRNA	miRbase ID	ThermoFisher ID	MiRbase sequence
let-7a-5p	MIMAT0000062	478575_mir	ugagguaguagguuguauaguu
miR-16-5p	MIMAT0000069	477860_mir	uagcagcacguaaauauuggcg
miR-107	MIMAT0000104	478254_mir	agcagcauuguacagggcuauca
miR-125a-5p	MIMAT0000443	477884_mir	ucccugagacccuuuaaccuguga
miR-139-5p	MIMAT0000250	478312_mir	ucuacagugcacgugucuccagu
miR-150-5p	MIMAT0000451	477918_mir	ucucccaacccuuguaccagug
miR-223-3p	MIMAT0000280	477983_mir	ugucaguuugucaaauacccca
miR-224-5p	MIMAT0000281	477986_mir	ucaagucacuagugguuccguuuag
miR-30b-5p	MIMAT0000420	478007_mir	uguaaacauccuacacucagcu
miR-335-5p	MIMAT0000765	478324_mir	ucaagagcaauaacgaaaaaugu
miR-342-3p	MIMAT0000753	478044_mir	ucucacacagaaaucgcacccgu
cel-miR-39-3p*	MIMAT0000010	478293_mir	ucaccggguguaaaucagcuug

*miRNA used for normalisation
